# Plio-Pleistocene deep-sea ventilation in the eastern Pacific and potential linkages with Northern Hemisphere glaciation

**DOI:** 10.1126/sciadv.add1467

**Published:** 2023-02-24

**Authors:** Liang Yi, Martín Medina-Elizalde, Liangcheng Tan, David B. Kemp, Yanzhen Li, Gunther Kletetschka, Qiang Xie, Huiqiang Yao, Huaiyu He, Chenglong Deng, James G. Ogg

**Affiliations:** ^1^ State Key Laboratory of Marine Geology, Tongji University, Shanghai, China.; ^2^ Department of Geosciences, University of Massachusetts, Amherst, MA, USA.; ^3^ State Key Laboratory of Loess and Quaternary Geology, Institute of Earth Environment, Chinese Academy of Sciences, Xi’an, China.; ^4^ Institute of Global Environmental Change, Xi’an Jiaotong University, Xi’an, China.; ^5^ State Key Laboratory for Biogeology and Environmental Geology and Hubei Key Laboratory of Critical Zone Evolution, School of Earth Sciences, China University of Geosciences (Wuhan), Wuhan, China.; ^6^ Institute of Hydrogeology, Engineering Geology, and Applied Geophysics, Faculty of Science, Charles University, Prague, Czech Republic.; ^7^ Geophysical Institute, University of Alaska Fairbanks, Fairbanks, AK, USA.; ^8^ Institute of Deep-sea Science and Engineering, Chinese Academy of Sciences, Sanya, China.; ^9^ Key Laboratory of Marine Mineral Resources, Ministry of Natural Resources, Guangzhou Marine Geological Survey, China Geological Survey, Guangzhou, China.; ^10^ Southern Marine Science and Engineering Guangdong Laboratory (Guangzhou), Guangzhou, China.; ^11^ State Key Laboratory of Lithospheric Evolution, Institute of Geology and Geophysics, Chinese Academy of Sciences, Beijing, China.; ^12^ University of Chinese Academy of Sciences, Beijing, China.; ^13^ Department of Earth, Atmospheric, and Planetary Sciences, Purdue University, West Lafayette, IN, USA.; ^14^ State Key Laboratory of Oil and Gas Reservoir Geology and Exploitation, Chengdu University of Technology, Chengdu, China.

## Abstract

Antarctic bottom water (AABW) production is a key factor governing global ocean circulation, and the present disintegration of the Antarctic Ice Sheet slows it. However, its long-term variability has not been well documented. On the basis of high-resolution chemical scanning of a well-dated marine ferromanganese nodule from the eastern Pacific, we derive a record of abyssal ventilation spanning the past 4.7 million years and evaluate its linkage to AABW formation over this period. We find that abyssal ventilation was relatively weak in the early Pliocene and persistently intensified from 3.4 million years ago onward. Seven episodes of markedly reduced ocean ventilation indicative of AABW formation collapse are identified since the late Pliocene, which were accompanied by key stages of Northern Hemisphere glaciation. We suggest that the interpolar climate synchronization within these inferred seven collapse events may have intensified global glaciation by inducing poleward moisture transport in the Northern Hemisphere.

## Teaser

The geochemical study of a ferromanganese nodule provides a 4.7-Ma record of abyssal oxygenation in the eastern Pacific.

## INTRODUCTION

Northern Hemisphere glaciation (NHG) initiated at the terminal Pliocene (~2.7 Ma ago) and underwent frequent alternations between glacial and interglacial states during the Pleistocene epoch (*1*, *2*). These glacial-interglacial cycles had a dominant 41-ka period in the early Pleistocene and shifted to a 100-ka period across the Mid-Pleistocene Transition (MPT; ~1.25 to 0.7 Ma ago), as reflected by marine foraminiferal oxygen isotope (δ^18^O) records (*1*, *2*). The amplitude of glacial-interglacial cycles increased after the MPT, and glacial cooling strengthened after the Mid-Brunhes Event (MBE; ~430 ka ago) (*1*, *3*). A number of hypotheses have been proposed to explain the onset of NHG, the MPT, and the MBE, including factors internal to the climate systems (e.g., ice volume dynamics and greenhouse gases) and external forcing (solar insolation variability) (*1*, *4*–*6*).

**Fig. 1.**
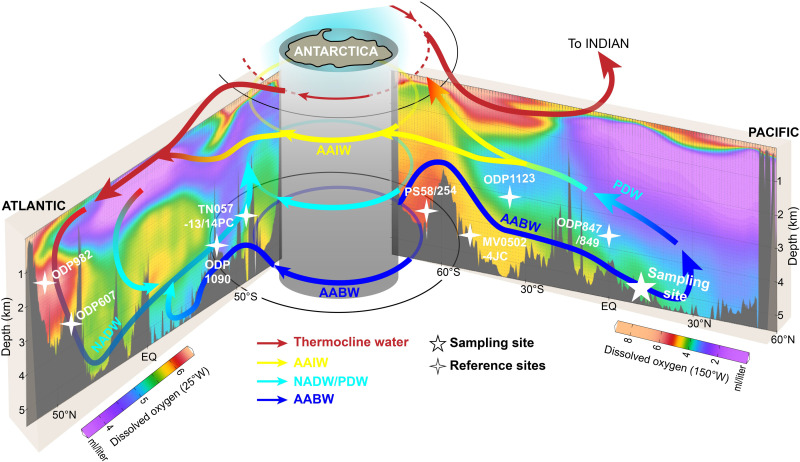
Schematic illustration of the circulation pattern in the Pacific and Atlantic Oceans, the vertical dissolved oxygen concentration distribution, and locations of the studied MFN (eastern Pacific) and sediment cores discussed in this paper. The ocean circulation pattern is modified from the work of Lumpkin and Speer (*79*). Dissolved oxygen data are from World Ocean Atlas 2013 (WOA2013) (*80*). Meridional overturning with circumpolar deep water (CDW) around Antarctica is not shown. AAIW, Antarctic intermediate water; NADW, North Atlantic deep water; PDW, Pacific deep water (low-oxygen layer).

Antarctic Ice Sheet (AIS) dynamics and associated climate variability likely contributed to the onset of NHG on various time scales (*7*, *8*). In particular, climate dynamics within the Antarctic region may have played a crucial role in modulating ocean and atmospheric CO_2_ reservoirs on glacial-interglacial time scales (*9*, *10*). Antarctic bottom water (AABW; Fig. 1) covers >70% of the total ocean-bottom region and 30 to 40% of the total global water mass (*11*). It has high dissolved oxygen concentration (*12*) and isolates CO_2_ in the deep ocean (*13*, *14*). The long-term evolution of AABW remains, however, poorly known. In this study, abyssal oxygenation in the deep Pacific, a proxy dominated by AABW dynamics (figs. S1 and S2), has been reconstructed across the past 4.7 Ma from geochemical analysis of a marine ferromanganese nodule (MFN). Our MFN metal-based redox record exhibits glacial-interglacial variability and suggests several intervals of low dissolved oxygen conditions that are indicative of collapses in AABW production. Our findings provide compelling evidence for a linkage between ephemeral episodes of low bottom-water oxygen concentration, AIS dynamics, and the evolution of NHG.

## RESULTS

### Age model

The studied MFN was collected from the Clarion-Clipperton (C-C) zone in the eastern Pacific (10.05°N, 154.32°W; 5050-m water depth). An existing time scale for the studied MFN (*15*) indicates that our chemical scanning (conducted at 5-μm intervals) provides an elemental abundance dataset with an average temporal resolution of 1.3 ± 1.0 ka (Materials and Methods; fig. S3).

Al and Mn abundances in the MFN exhibit dominant variability at 100- and 405-ka periods (fig. S4). Al is closely associated with eolian dust in deep-sea sediments in the North Pacific (*16*). In the studied MFN, Al is embedded between Mn and Fe layers or scattered randomly (fig. S5, B and C), indicating that Al does not accompany Mn-Fe but was captured during MFN growth, similar to other MFN and marine ferromanganese crust (MFC) regions (*17*). The captured debris in the studied MFN has a similar composition of rare earth elements (REEs) to samples from the Chinese Loess plateau (fig. S5D), inferring the MFN Al content as a proxy of eolian dust. Mn and other metals, such as Ni and Cu, are sensitive to changes in seawater dissolved oxygen (*17*, *18*) because they are usually dissolved from the oxygen minimum zone (OMZ), and their oxides favor an oxidized environment below the OMZ (*19*).

The existing age model (*15*) can be refined by tuning the Al and Mn data to orbital eccentricity (*20*) based on the observed elemental variability resembling eccentricity (fig. S6), with the hypothesis that eccentricity was the dominant factor mediating eolian dust input in the Plio-Pleistocene epochs (*21*, *22*). Subsequently, three MFN time scales have been developed for the studied MFN to assess the robustness of paleoenvironmental inferences (figs. S3 and S4). Age uncertainties in these models are relatively small (errors 25 ± 14 ka up to 58 ka) during the studied growth period (4.7 Ma).

### MFN geochemistry as a water-mass proxy

Hydrogenic and diagenetic MFC/N can provide a direct record of oceanographic change (*23*). For example, MFC/N may form via the precipitation of metal oxide in water under oxic conditions (*24*), and metal oxides are the primary component of MFC/Ns (*25*). The accumulation of redox-sensitive metals in MFC/N means that they can be sensitive recorders of bottom-water redox variations, while the potential relationship between them has yet not been fully understood.

In the C-C zone, marine productivity is relatively low (fig. S1A) (*26*), and bottom ventilation (i.e., redox conditions) is dominantly controlled by the intensity of AABW formation (figs. S1C and S2) (*27*–*29*). The utility of MFNs in the C-C zone as archives of bottom ventilation and AABW formation is underlined by the fact that they mainly form by hydrogenic and diagenetic processes (*30*) without influence from hydrothermal process (fig. S5F). In addition, old MFNs in the C-C zone (formed in the Oligocene and Miocene) and their fragments could be an important polymetallic source for the formation of more recent MFNs (i.e., the Plio-Pleistocene) via (re)generation processes (*31*). Hence, it is unlikely that the seawater metal composition was a limiting factor controlling nodule growth in the C-C zone.

Moreover, during the past century, the association between bottom-water fluxes across the geographic sector 150°W, 0° to 20°N and the Samoan passage is significant (*r* = 0.32, *P* < 0.01; fig. S2), and the latter is the main pathway of AABW from the Southern Ocean entering the North Pacific (*32*). This relationship further supports the notion that AABW variability is the major factor controlling ventilation beneath the Eastern Tropical Pacific.

Previous studies have proposed that Mn variability is positively associated with changes in dissolved oxygen (*17*, *24*) because of the migration of oxygen-sensitive (OS) metals from reducing to oxidizing environments (*27*). Notably, there is a statistically significant correlation (*P* < 0.01) between Mn and S and between Fe and S (fig. S7, A and B), consistent with the interpretation of MFC/N being recorders of bottom-water redox variations (*23*), because S presents mainly as sulfides (*33*). The observed data scattering may reflect (i) a mixture of sulfate and sulfide in our S data and/or (ii) that different types of nodule growth may affect MFN elemental composition (*17*).

Comparisons of MFN metal records, including Ni, Mn, and Cu, to various proxies of deep-sea ventilation from different sites support our inference (fig. S7, C to F, and table S1). Specifically, we observe synchronous changes in MFN elemental content data (Ni, Mn, and Cu) and the benthic foraminiferal δ^13^C record of core MV0502-4JC from the Southern Ocean (fig. S7E) (*34*). The benthic foraminiferal δ^13^C record is interpreted to reflect changes in lower circumpolar deep water (LCDW) intensity (*34*) because different water masses have distinct δ^13^C values (*35*). A comparison between our MFN metal records and the sediment grain size record from Ocean Drilling Program (ODP) site 1123, located northeast of the Chatham Islands, records of the Pacific deep western boundary current (*36*), also highlights similar variations (fig. S7D). Larger grain sizes at ODP site 1123 reflect intensified near-bottom flow vigor and better ventilation in the Southern Ocean (*36*). In addition, an antiphase relationship is observed between the nodule elemental records and the core TN057-13/14PC authigenic uranium record from the Southern Ocean (fig. S7C), a record expected to reflect deep-sea oxidation (*37*) because uranium generally becomes enriched under reducing conditions (*17*, *18*).

The above comparisons support the interpretation that the MFN metal composition (Ni, Mn, and Cu) represents a proxy for dissolved oxygen and bottom ventilation in the C-C zone. Leveraging on the high consistency among Ni, Mn, and Cu (fig. S8), we use the arithmetic mean of these three metals (standardized) as a proxy for the evolution of bottom-water redox conditions (OS index). Our OS index, with an average value 0.32 ± 0.14, inherits the variability of the three metals (fig. S9), and thus, high-MFN metal contents are represented by high OS index values. In turn, high OS index values reflect enhanced ventilation and higher dissolved oxygen. Similar variability to the OS index that is based on metal ratios instead can be observed (fig. S10), emphasizing that the influence of different types of nodule growth and potential influence on elemental composition and the OS index is likely negligible and that the data scatter in fig. S7 (A and B) may be attributable instead to the presence of both sulfate and sulfide in chemical scanning.

**Fig. 2.**
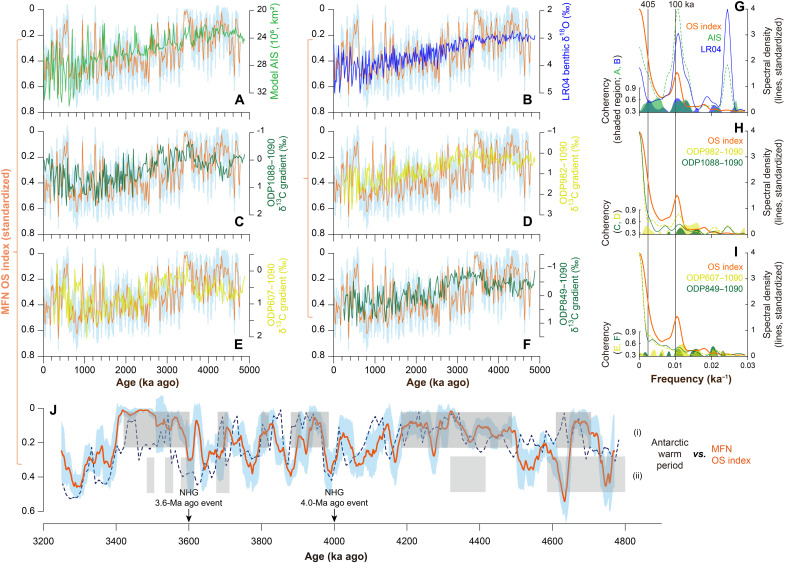
Comparison between MFN OS index and various water-mass proxies. (**A**) AIS, the modeled West AIS (*64*), derived as a function of LR04 record and local solar insolation. (**B**) The LR04 benthic δ^18^O stack (*3*), indicating variability of Northern Hemisphere (NH) ice volume and temperature. (**C** to **F**) Various water-mass proxies inferred from the gradient between benthic foraminiferal δ^13^C records (*38*). The MFN OS index is developed in this study and shown with the 68% confidence interval [light blue shaded region in (A) to (F) and (J)]. (**G** to **I**) Cross-spectral comparisons between the paired data in (A) to (F) using the ARAND package (*81*). Coherence spectra above the 80% confidence level are shown (any coherence data <80% are not plotted). The correlation coefficient of these comparisons can be found in table S2 (see also fig. S11). (**J**) Warm periods (gray bars) around Antarctica, identified from (i) Pliocene high-productivity intervals at IODP site U1361 (*41*) and (ii) Diatom and silicoflagellate assemblages from the Kerguelen plateau (*82*) and Prydz Bay (*83*). Two NHG events that occurred at 3.6 and 4.0 Ma ago are identified from both marine and terrestrial records (*3*, *55*). The normalized AIS (A) is also shown in (J) as a reference (dash line).

Further support for the interpretation of the OS index as a record of deep-sea oxygenation is provided by the observed correlation between the OS index, the identified warm periods, and basinal gradients in benthic foraminiferal δ^13^C (Fig. 2, fig. S11, and table S2), broadly indicating the intensity of deep circulation in the Atlantic and Pacific (*38*). The moderate correlation between them suggest, however, potential uncertainties in proxy interpretation, like the influence of multiple sources of δ^13^C affecting the interpretation of benthic foraminiferal δ^13^C as a proxy of water masses (*35*) or the supply of metals and different types of nodule growth affecting MFN elemental composition (*17*), in addition to chronological and analytical uncertainties associated with these paleoclimate records. A weak relationship between the MFN OS index and marine productivity proxies at ODP site 846 (*39*) and site 849 (*40*) in the eastern Pacific are observed (table S2), indicating that the degradation of organic matter and consumption of dissolved oxygen may have little influence on abyssal oxidation in the C-C zone.

**Fig. 3.**
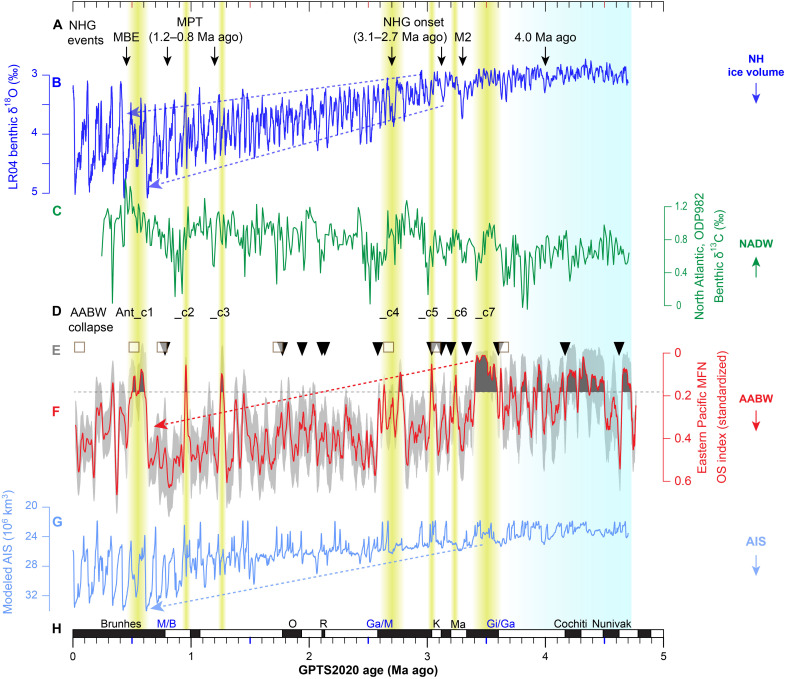
Abyssal ventilation in the eastern Pacific during the Plio-Pleistocene epochs and the potential collapse events. (**A**) Key stages in NHG evolution (intensification) summarized from previous studies (*3*, *55*). The light blue shaded interval referenced to Fig. 2J. (**B**) Benthic δ^18^O stack LR04 (*3*). (**C**) ODP site 982 benthic δ^13^C (*38*) from the North Atlantic (57.31°N, 15.53°W; 1145-m water depth), a proxy for upper NADW intensity (Fig. 1). (**D**) Inferred ventilation collapse events in AABW formation as recognized in this study are highlighted with yellow shaded intervals. A collapse event is defined by OS index values <0.18, which is the lower boundary of the 68% confidence interval (0.18, 0.46) of our OS index. These events are evident in all three time scales of this study (table S3). (**E**) Paleomagnetic (triangles) and ^10^Be/^9^Be (squares) age control points for the studied MFN (*15*). (**F**) The OS index of the studied MFN (this study) smoothed by a 21-ka moving average. The gray shaded region represents the 68% confidence interval. (**G**) Modeled West Antarctic ice volume (*64*). Arrows in (F) and (G) indicate that the amplitude of AABW variability and modeled West AIS have both decreased substantially since 3.4 Ma ago. (**H**) Geomagnetic Polarity Time Scale (GPTS2020) (*84*). M/B, Matuyama-Brunhes boundary; O, Olduvai; R, Réunion; Ga/M, Gauss-Matuyama boundary; K, Kaena; Ma, Mammoth; Gi/Ga, Gilbert-Gauss boundary. Arrows on the right panel show direction of enhancement of various processes.

**Fig. 4.**
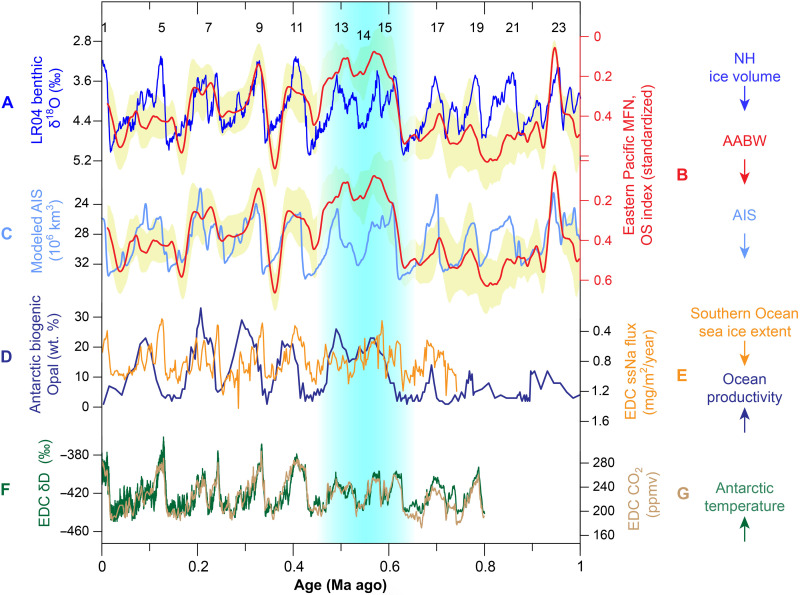
Comparison between various proxies over the past 1 Ma. (**A**) The LR04 benthic δ^18^O stack (*3*), indicating variability of NH ice volume and temperature, with labeled MIS 1 to 23. (**B**) OS index of the studied MFN smoothed using a 21-ka moving average (this study), positively correlated with abyssal oxidation. The yellow shaded region represents 68% confidence interval. (**C**) Modeled West Antarctic (Pacific-sector) ice volume (*64*), derived from a function of LR04 record and local solar insolation. (**D**) Biogenic opal content that indicates biological productivity from Antarctic PS58/254 site (*47*); see site location in Fig. 1. (**E**) EPICA Dome C (EDC) ice core sea-salt Na flux (ssNa), which is an indicator of Southern Ocean sea ice extent (*54*). (**F**) EDC ice core δD record, which mostly reflects Antarctic air temperature variability (*53*). (**G**) EDC ice core CO_2_ record (*85*). The blue shaded area marks the extra-long interglacial MIS 15 to 13 discussed in the main text. Arrows on the right panel indicate direction of enhancement of various processes.

Integrating all these observations, we propose that the OS index reflects oxygenation variability at the site during the Plio-Pleistocene and that this variability was controlled primarily by shifts in the strength of LCDW/AABW formation, notwithstanding possible secondary influences on redox element abundance. Last, improved consistency in proxy comparisons among the three age models derived for the MFN OS index (table S2) enables using the Mn-based tuning time scale for further paleoenvironmental inference (Figs. 3 and 4).

## DISCUSSION

### Plio-Pleistocene changes in abyssal ventilation

Using our MFN OS index, it is possible to trace the history of abyssal ventilation back to 4.7 Ma ago, which highlights the consistency between various water-mass proxies on eccentricity time scales (~80 to 120 ka and ~405 ka; Fig. 2, G to I, and fig. S11). During the early Pliocene (ca. 4.7 to 3.4 Ma ago), both NHG and abyssal ventilation in the eastern Pacific were substantially amplified (Fig. 3, B and F), in agreement with Pliocene West AIS oscillations (Fig. 2J and fig. S7G) (*41*). Moreover, linear trends toward lower MFN OS index (Fig. 3F) and increased modeled West AIS maxima (Fig. 3G) are distinctive features from the late Pliocene onward. These trends from ~3.4 Ma ago onward accompany NHG enhancement at ~3.15 Ma ago (Fig. 3B), in agreement with prolonged surface water cooling around Antarctica since ~3.3 Ma ago (*7*). This implies that AABW influence in the eastern Pacific and AIS variability closely coevolved until they reached a relatively stable long-term state after the MPT.

Detailed analysis of data across the past 1.0 Ma demonstrates that the MFN OS index (Fig. 4B) and modeled West AIS variability show substantial covariation with each other (Fig. 4C) and with Northern Hemisphere high-latitude climate records, on glacial-interglacial time scales (Fig. 4A). Positive correlation between the OS index, modeled West AIS, and ice volume variability suggests enhanced deep sea oxygenation at the nodule site during times of increase AIS and Northern Hemisphere ice volume extent. During glacial states, enhanced AABW production and flow into the Southern Ocean would increase abyssal ventilation in the eastern Pacific, as reflected by the OS index record, ultimately increasing the relative volume of AABW in the deep ocean and CO_2_ sequestration (*42*–*44*) and further strengthening glacial conditions (and vice versa during interglacial states; Fig. 4G). This mechanism links glacial expansion of southern-sourced water into the North Atlantic (*45*) and the alternation of greenhouse gases (between interglacial outgassing to the atmosphere and glacial storage in the deep ocean) by changing stratification in the Southern Ocean (*9*, *10*). During periods when North Atlantic deep water (NADW) formation strengthened (Fig. 3C), as indicated by benthic foraminiferal δ^13^C data from the North Atlantic (*38*), lower OS index values reflect lower oxygen conditions during some key intervals, such as at ~0.95 Ma ago [marine isotope stage 23 (MIS 23)] and 0.62 to 0.48 Ma ago (MIS 15 to 13). This evidence suggests an inverse relationship between NADW and abyssal ventilation in the eastern Pacific at times, in agreement with the bipolar seesaw model for AABW production hypothesized for the last deglaciation (*46*) and for the past glacial-interglacial alternations (*36*, *45*).

During MIS 15 to 13 (ca. 0.6 to 0.5 Ma ago), the MFN OS index notably decreased (OS < 0.18), suggesting that the AABW influence over the abyssal Pacific may have been greatly reduced at this time (Fig. 4B). Hence, this potentially signals an AABW collapse event. High biological productivity and lithogenic sediment supply identified in core PS58/254 out of the Pine Island Bay suggest that MIS 15 to 13 was the most likely period for a disintegration of the West AIS during the past 0.8 Ma (Fig. 4D) (*47*). Climate models have shown that an event of AIS disintegration can thicken and increase the extent of Southern Ocean sea ice, inhibit AABW formation, warm the deep ocean, cool the ocean surface, and ultimately lead to Southern Hemisphere cooling (*48*, *49*). The influence of a Southern Ocean sea ice positive anomaly can propagate globally via atmospheric Rossby waves (*50*), such as what may have happened during the extra-long interglacial period in the eastern Pacific (*51*) and in the Chinese Loess plateau (*52*) during MIS 15 to 13. Thus, integrating relatively low interglacial temperatures as reflected by Antarctic ice core temperature records during MIS 15 to 13 (Fig. 4F) (*53*) with relatively enhanced Southern Ocean sea ice extent during MIS 14 to 13 (Fig. 4E) (*54*), we propose that an AIS disintegration during 0.6 to 0.5 Ma ago might have produced a large amount of sea ice, inhibiting CDW mixing with ice shelf water, and ultimately preventing AABW formation and abyssal ventilation in the eastern Pacific.

Inferred intervals of poor abyssal ventilation in the eastern Pacific are observed not only during the Mid-Pliocene (Fig. 2J) and MIS 15 to 13 but also within several parts of the Pleistocene (Fig. 3F). This suggests further intervals when the AABW influence in the eastern Pacific might have been reduced to a much lower level. These episodes are characterized by OS values <0.18, defined here as potential collapse events. Seven collapse events are labeled as follows: Ant_c7 to Ant_c1 and are dated here at 3.6 to 3.4, ~3.2, ~3.0, 2.8 to 2.6, 1.3 to 1.2 (MIS 42 to 38), ~0.95 (MIS 23), and 0.62 to 0.48 Ma ago (MIS 15 to 13), respectively (Fig. 3D). These events are evident in all three MFN time scales (table S3). All seven events slightly preceded or accompanied key stages in the progression of NHG (Fig. 3A), which have been previously identified from both marine and terrestrial records at 3.3 Ma ago (M2 event), 3.15 and 2.74 Ma ago (the onset of NHG), 1.25 and 0.7 Ma ago (MPT), and 0.45 Ma ago (MBE) (*1*, *3*, *55*). However, the phase difference between NHG and potential collapse events of abyssal ventilation in the eastern Pacific is not consistent, which might be related to age uncertainties. For example, Ant_c6 preceded the early onset of NHG at 3.15 Ma ago, constrained by geomagnetic chron C2An.2n (Fig. 3H), agreeing well with previous studies (*7*, *8*), whereas Ant_c5 appeared to be more synchronous with the NHG event at 2.74 Ma ago. Considering that the key transitions in NHG evolution were not abrupt but generally gradual, together with age uncertainties in the MFN time scales, we propose that NHG establishment during the Plio-Pleistocene epochs was persistently accompanied by Antarctic climate variability and shifts in AABW production.

### Possible mechanism of the collapse events

**Fig. 5.**
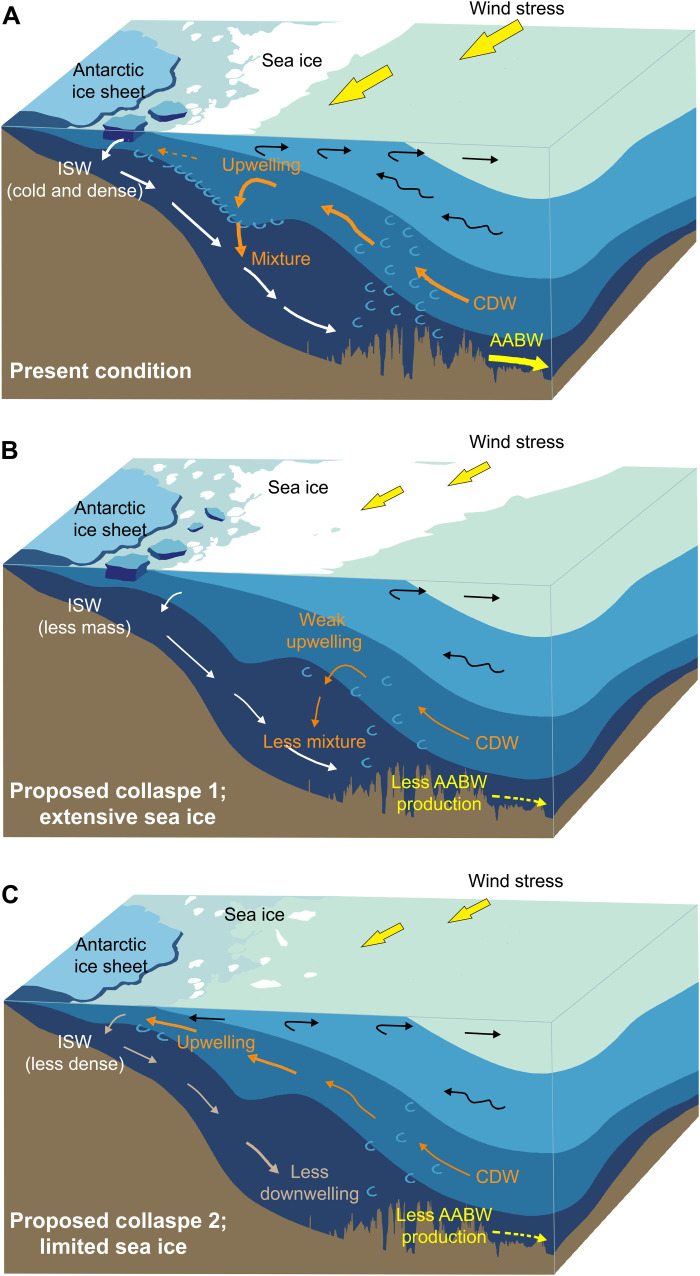
Schematic illustration of AABW production and proposed collapse mechanisms. (**A**) At the present day, AABW forms when sea ice and brine production create high-salinity water, which, in turn, interacts with ice shelves to form ice shelf water (ISW). The resulting dense and ice water mixture travels across the continental shelf and sinks to depth. (**B**) Glacier collapse and extensive sea ice cover can prevent AABW production by blocking wind stresses on the ocean surface, weakening upwelling processes, and thus reducing the supply of CDW (*63*). (**C**) When the sea ice cover is limited, the production of dense, high-salinity ISW is reduced, which allows CDW upwelling onto the Antarctic continental shelf, reducing AABW formation (*7*, *66*). The basic map was modified from (*86*).

AABW forms when brine rejection associated with sea ice production creates dense high-salinity water (Fig. 5A), which, in turn, interacts with ice shelves to form ice shelf water (*56*). The resulting dense and cold water masses travel along the continental shelf and sink to depths of 2000 to 4000 m (*57*). Modern observations suggest that AABW formation is related to Antarctic sea ice production during the austral winter (*58*) and with the supply of CDW (*59*) induced by surface winds that enhance salinity and fluxes of ice shelf water (*60*–*62*). Moreover, the present AIS disintegration is observed to prevent AABW production by reducing the supply of CDW (*63*). These modern observations and modeling studies help explain the consistency between the OS index and modeled West AIS variability (*r* = 0.42 to 0.53, *P* < 0.01) (*64*) and the stacked marine benthic foraminiferal δ^18^O record (*r* = 0.50 to 0.57, *P* < 0.01; table S2) (*3*) and suggest two end-members of AABW formation: one, via wind-induced upwelling of CDW and, another, via density-induced downwelling of ice shelf water (Fig. 5A).

On the basis of the processes involved in AABW formation described above (*56*, *57*, *65*) and the proposed linkage between the OS index and LCDW/AABW formation (Figs. 3 and 4), we suggest that the seven episodes of ventilation collapse (Ant_c1 to c7) identified in the OS index were associated with changes in AABW production and AIS extent, although their underlying driving mechanisms may not have been the same (Fig. 5). For example, during MIS 15 to 13, as described in the previous section, extensive sea ice cover likely prevented surface winds inducing CDW upwelling that mixed with ice shelf water, thus reducing AABW production (Fig. 5B). On the other hand, if sea ice cover was extremely limited, then it would allow warm CDW upwelling onto the Antarctic continental shelf, preventing downwelling of lower density ice water to form AABW (Fig. 5C). The latter scenario may have been the case for the Mid-Pliocene warm period (*7*, *66*) and the last deglaciation (*49*, *67*) and has been reproduced in the Hadley Centre coupled ocean-atmosphere climate model for the Plio-Pleistocene transition (*68*).

Considering that large amounts of moisture are needed to fuel NHG intensification (*69*), evidence of AABW production collapse events coeval with NHG suggests a potential mechanism involving warm Antarctic climate in intensifying NHG. We hypothesize that when AABW production collapsed (Ant_c1-c7), a reduced AIS might have freshened Antarctic intermediate water and induced North Atlantic surface waters to sink deeper (*70*), thus enhancing NADW formation and Atlantic meridional overturning circulation (AMOC) during these periods (Fig. 3C). Consequent enhanced AMOC transported large amounts of moisture from the tropics to the northern high latitudes (*71*), fueling NHG intensification (*72*). Furthermore, ice melting in the Southern Ocean absorbed large amounts of heat, which ultimately helped Antarctic climate to return to a glacial state (*48*). One such example likely occurred before the end of the MPT (*6*, *73*), at ~0.9 Ma ago, during the Ant_c2 event. In addition, final closure of the Panama Isthmus during the onset of NHG probably prevented the northern high latitudes from returning to the warm climate state of the Middle Pliocene (*71*, *74*). Thereafter, NHG, AIS, and AABW were substantially enhanced, as reflected by the long-term trend in all proxy records (Fig. 3). Our reconstruction of abyssal ventilation in the eastern Pacific, therefore, provides evidence for a close coupling between AABW ventilation and NHG, likely via a common forcing from the AIS.

## MATERIALS AND METHODS

The C-C zone in the eastern Pacific (fig. S1A) is located north off the equatorial high-bioproductivity zone (*75*) and is known as the global “manganese nodule belt” (*76*). Because of the influence of AABW currents in the C-C zone since the late Oligocene (*27*), abyssal erosion has occurred on a large scale, facilitating the abundant development of MFNs on the sea floor (*77*). Formation of MFCs or MFNs on the deep-sea floor results from the migration of OS metals from reducing to oxidizing environments (*27*). Because of their continuous but slow growth (usually <1 cm/Ma), MFC/N can record deep-sea geochemical changes, providing an opportunity to trace the long-term evolution of AABW formation (*27*).

The studied MFN was collected from the C-C zone (10.05°N, 154.32°W; 5050-m water depth) in 2013 using a box corer on cruise DY125-27 (R/V HAI YANG LIU HAO). The vertical direction of the sample was identified in the box corer and verified in the laboratory by its smoother surface at the top with some white and white-gray benthos. It is brownish-black in color and with a densely packed, laminated growth pattern. It was cut into three pieces, and the middle part was polished on one side for the chemical scans (36-mm length, 24-mm width, and 5-mm thickness).

For precise dating, Yi *et al.* (*15*) developed a new method based on an integration of high-resolution magnetic scanning and ^10^Be dating. The method can be used at a resolution up to 80 μm. Thus, integration of magnetic scanning, authigenic ^10^Be/^9^Be, and the Co flux chronometer has allowed a robust geochronological framework to be established for the studied MFN (*15*). The existing geochronology reveals continuous deposition from 4.70 ± 0.15 Ma ago to the present. Variations in Mn/Fe suggest that MFN growth resulted from a combination of hydrogenetic and diagenetic processes (*15*) and that changes in abyssal redox condition were the key factor governing MFC/N growth (*27*). The dating results are displayed in fig. S3 and table S4.

To determine environmental details during nodule growth in this study, chemical scanning was conducted at 5-μm interval on the left side of the middle part of the studied MFN using a VEGA 3XM TESCAN instrument (EDAX) at the Institute of Geology, Czech Academy of Sciences (17.96-mm length and 3595 data points in total). Before analysis, the thin sample was placed in the vacuum chamber of the electron-probe microanalyzer, and the air contained within the pores of the nodule was driven out using a vacuum pump. The accelerating voltage, beam current, and an analysis spot diameter were set as 20 kV, 2 × 10^−8^ A, and 5 μm, respectively, with an SD of the identified element of <1.5% over the period of measuring. Elements including Ni, Mn, Cu, Co, Fe, Ti, Al, Cl, and Si are quantified as atomic percent.

To verify the linkage between eolian particles in the studied MFN and Chinese loess, two samples were collected during cutting for REE analysis. The two samples were firstly leached with 20 ml of 1 mol/L hydrochloric acid (HCl) for 24 hours at 50°C, following the method of Wei *et al.* (*78*). After leaching, the residues of the leached samples were completely digested with concentrated HF-HNO_3_-HClO_4_ in an airtight Teflon container. REE concentrations were determined by inductively coupled mass spectrometry (PQ3, Thermo Elemental) at the State Key Laboratory of Marine Geology, Tongji University.

## Supplementary Materials

### This PDF file includes:

Figs. S1 to S11 Tables S1 to S5 References
