# Supplementary material for: Plio-Pleistocene deep-sea ventilation in the eastern Pacific and potential linkages with Northern Hemisphere glaciation
Source: Sci Adv. 2023 Feb 24;9(8):eadd1467. doi: 10.1126/sciadv.add1467 (PMC9956117; doi:10.1126/sciadv.add1467)
Supplement: Supplementary file 1 — Figs. S1 to S11 Tables S1 to S5 References [file sciadv.add1467_sm.pdf]

Science Advances
AAAS

Supplementary Materials for

Plio-Pleistocene deep-sea ventilation in the eastern Pacific and potential linkages with Northern Hemisphere glaciation

Liang Yi *et al.*

Corresponding author: Liang Yi, yiliang@tongji.edu.cn; Huiqiang Yao, hqyao@163.com; Chenglong Deng, cldeng@mail.iggcas.ac.cn

Sci. Adv. **9**, eadd1467 (2023)
DOI: 10.1126/sciadv.add1467

This PDF file includes:

Figs. S1 to S11
Tables S1 to S5
References

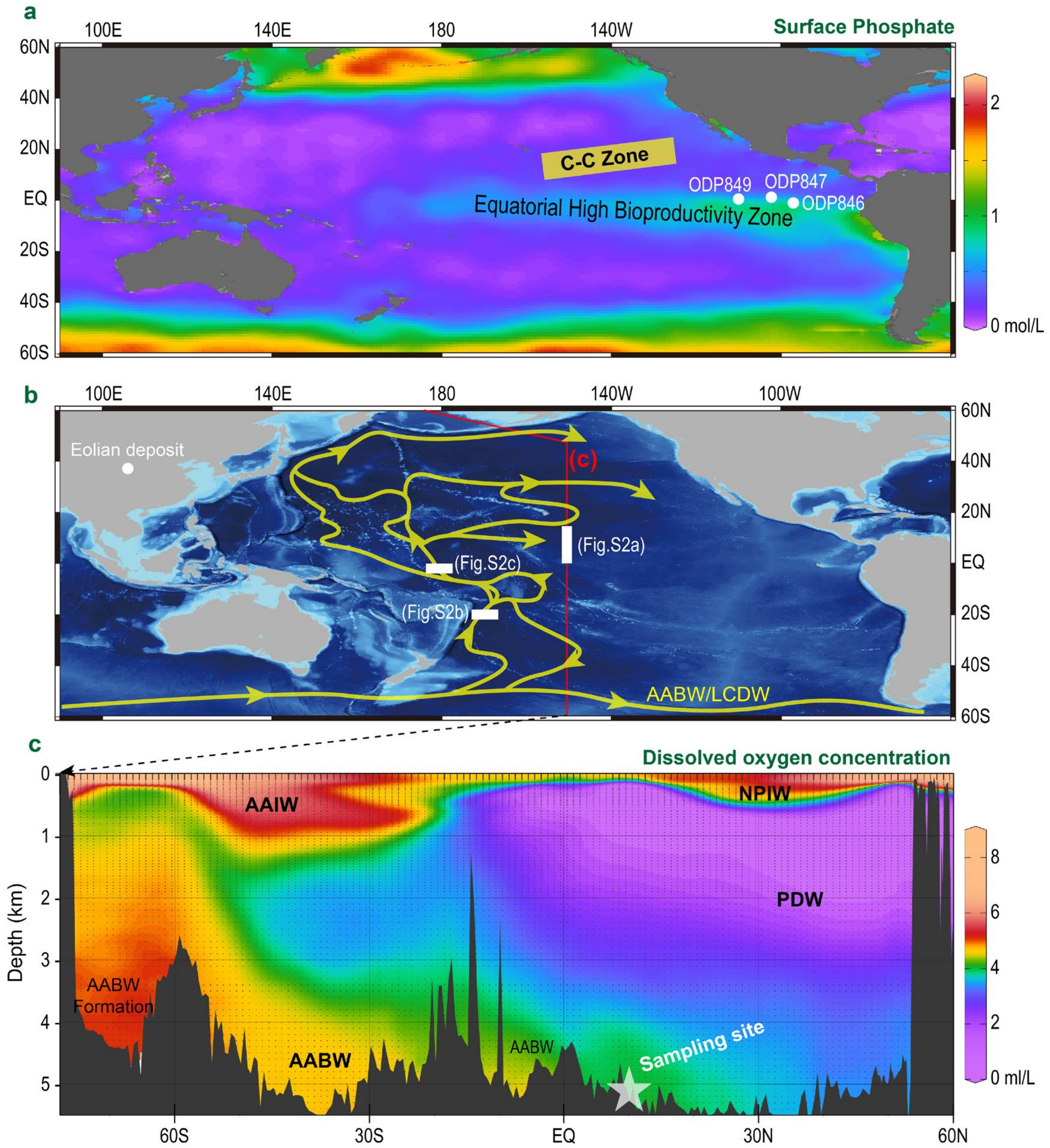

Fig. S1 Oceanographic map of the Pacific. a, Surface distribution of phosphate in the Pacific. Clarion-Clipperton Zone (C-C Zone); the Equatorial high bioproductivity zone is shown on a map of phosphate content (*75*). **b,** Bottom water circulation in the Pacific Ocean (*32*). AAIW, Antarctic intermediate water; AABW, Antarctic bottom water; LCDW, lower Circumpolar deep water; NPIW, North Pacific intermediate water; PDW, Pacific deep water (the low oxygen and high nutrient layer in the Pacific Ocean). The white dots in (**a-b**) are sites mentioned in the main text and used for

comparison. **c,** The average dissolved oxygen concentration data along the 150°W transect. Data presented in (**a**) and (**c**) are from World Ocean Atlas 2013 (WOA2013) (*80*).

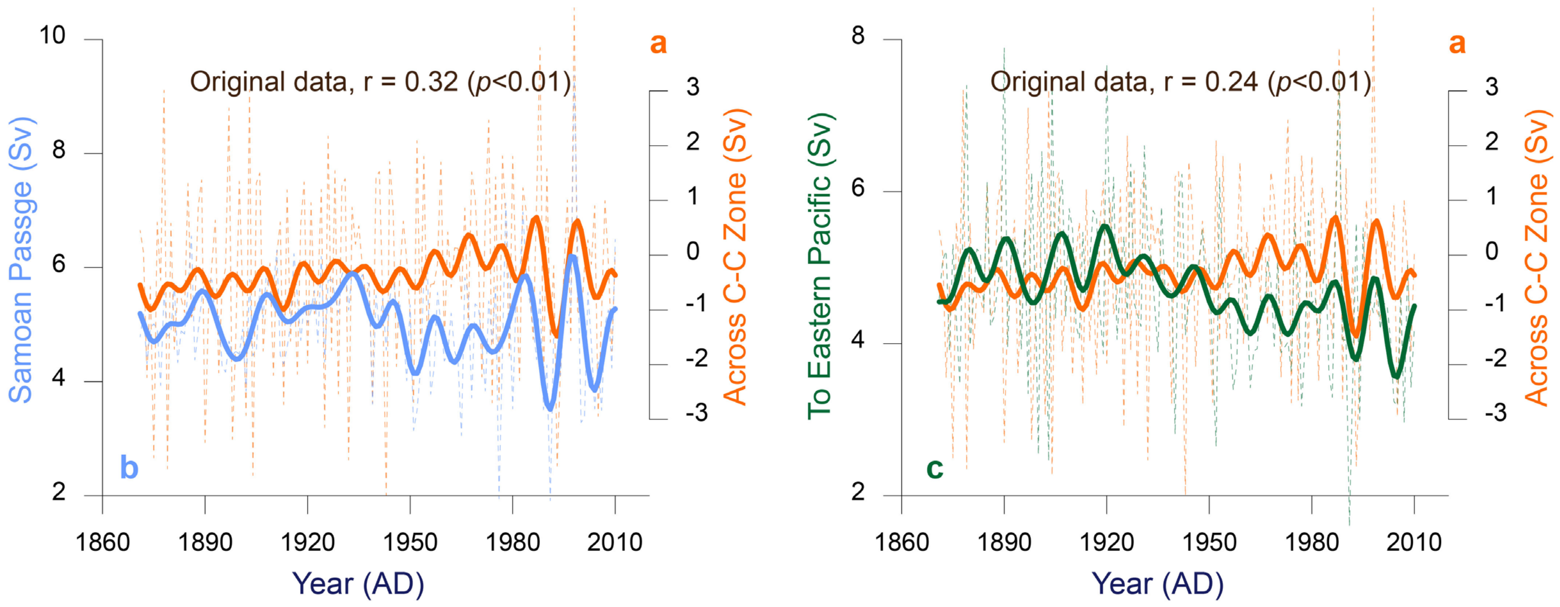

Fig. S2 Annual changes in AABW transport in the Pacific. a, Bottom water across the C-C Zone (profile line: 150°W, 0–20°N, below 4000 m). **b**, AABW transport by the Samoan Passage. **c**, AABW transport to the Eastern Pacific (profile line: 175–185°E, 0°, below 4000 m). Dashed lines, original data; Bold lines, FFT-filtered low-frequency (< 0.1) band-passed variation. The Samoan Passage is the main entrance of AABW from the Southern Ocean into the North Pacific (*32*). The bottom water flux was derived from the Simple Ocean Data Assimilation (SODA version 2.2.4; 1871–2010 AD) (*87, 88*), consisting of gridded variables for the global ocean.

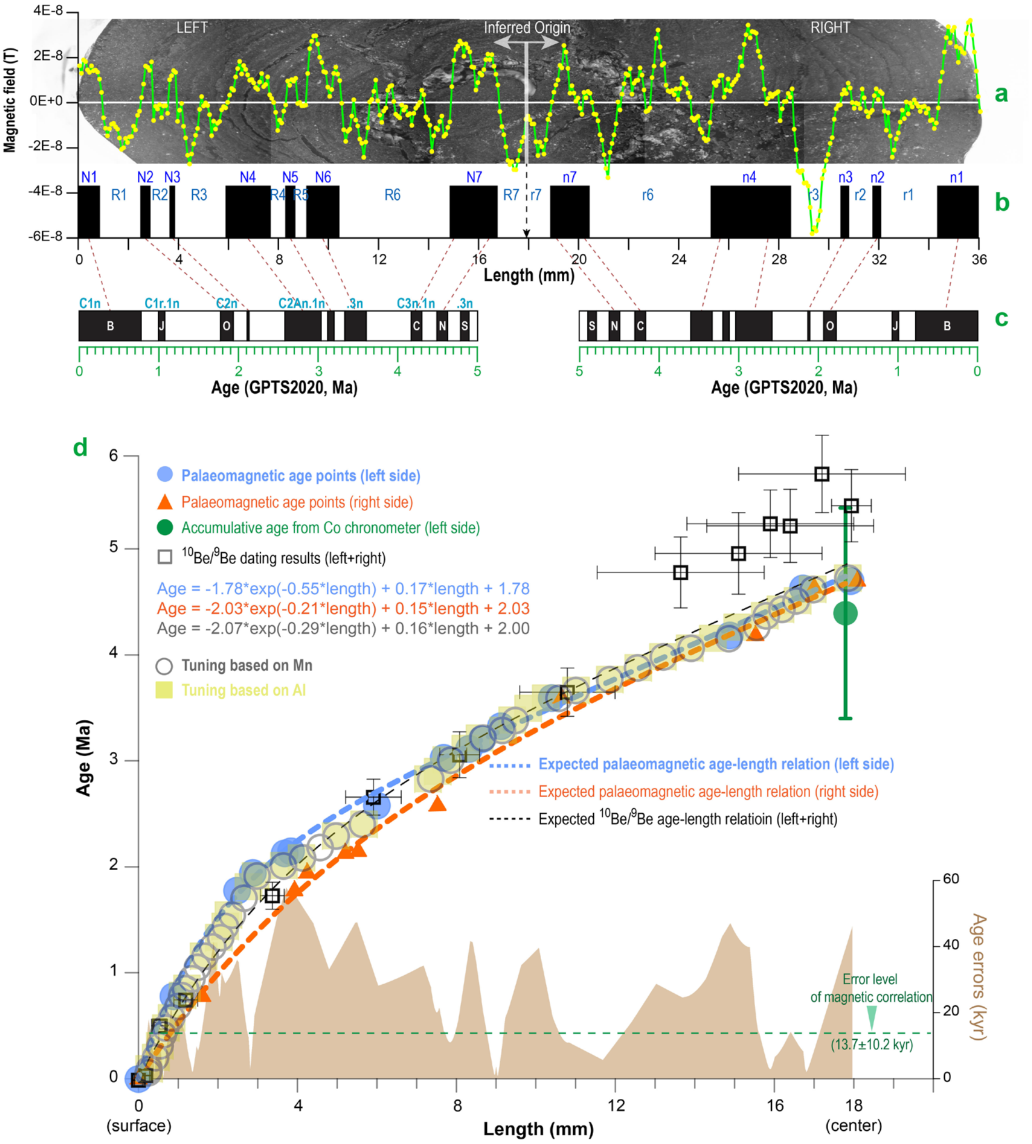

Fig. S3 The polished side of the studied MFN and three age models. a, the magnetization record of the studied MFN determined by magnetic scanning with a photo of the polished side. **b,** Normal and reversed magnetozones (24 in total) are numbered N1–N7 and n1–n7, and R1–R7 and r1–r7, respectively (uppercase and lowercase denote the left and right sides of the nodule, respectively). **c,** The geomagnetic polarity timescale (GPTS2020) (*84*). B, Brunhes; J, Jaramillo; O, Olduvai; C, Cochiti; N, Nunivak; S, Sidufjall. Dashed lines show correlations to GPTS2020. **d,** Age models and error for the studied MFN. Noted that age errors were

the standard deviation of three age models at each point.

The geochronological framework and growth rates were established by linear interpolation of paleomagnetic constraints. All parameters of the three formulae (dash lines) are significant at $p < 0.1$ level with > 99% of the total variance explained, demonstrating continuous growth of the studied MFN and consistency between paleomagnetic and $^{10}Be/^{9}Be$ dating. This age model indicates average growth rates of 4.20 ± 2.21 mm/Myr and 4.94 ± 4.15 mm/Myr for the left and right sides, respectively. The reliability of the MFN geochronology has been verified by comparing all age constraints, the expected age-length relationship, and the expected starting growth age resulting from various dating methods, respectively (*15*). Based on these age constraints, the age model on the left side was slightly modified in this study by tuning MFN Mn and Al contents to Earth's eccentricity (table S4).

For the tuning processes, the Mn and Al records and Earth's eccentricity were visually matched (fig. S6), then the MFN elemental time series was filtered using a band-pass filter centered on the eccentricity frequency (80-120 kyr), and the resulting curves were correlated with the unfiltered eccentricity time series to assess its robustness. Additional age control points were added iteratively until the unfiltered MFN elemental time series and the eccentricity time series showed a good match (fig. S6). In order to test the coherency between the MFN elemental records on the new chronology and Earth's eccentricity time series, spectral analyses were conducted (fig. S4). Results indicate that, at eccentricity periodicities, i.e. ~405 kyr and ~100 kyr, the coherency is significant ($p < 0.05$). The correlation coefficients between the eccentricity time series and the elemental variation were also notably improved after tuning (table S2).

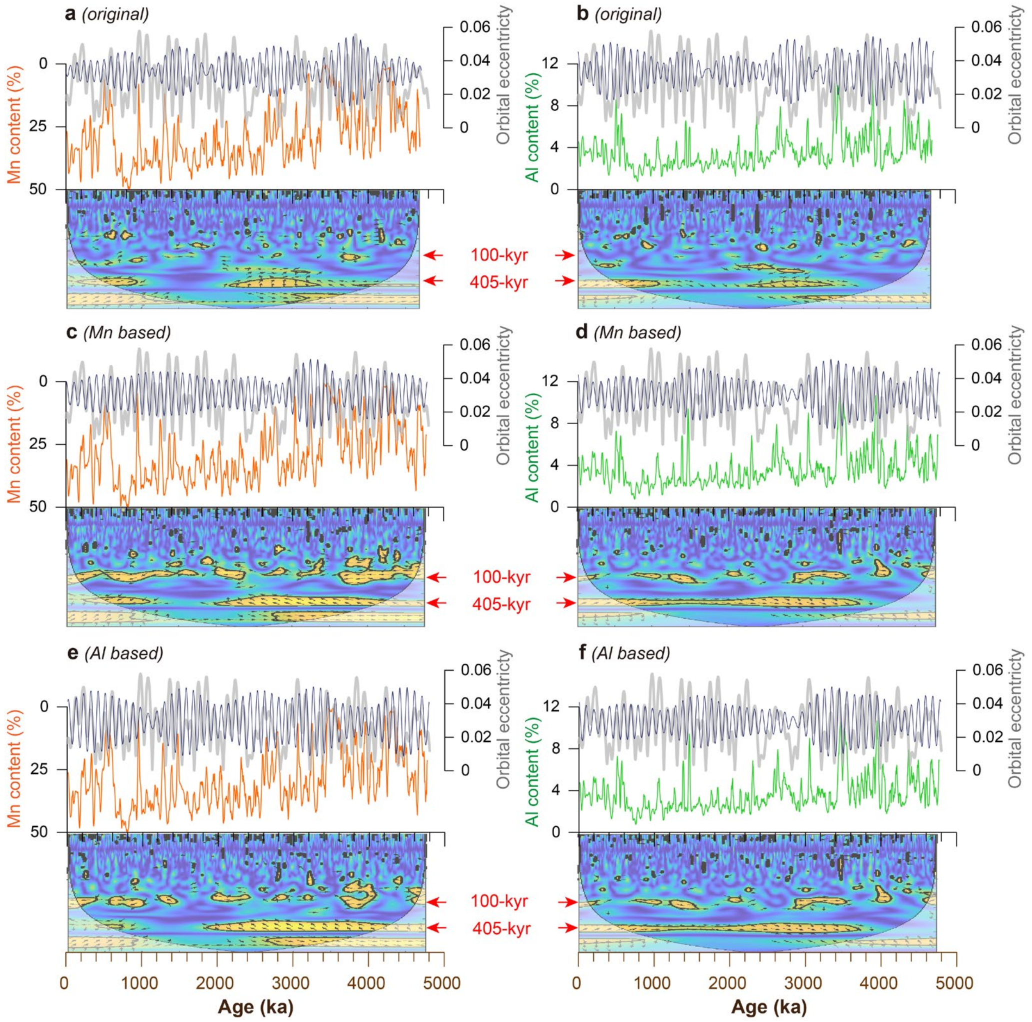

Fig. S4 Testing the relationship between MFN and Earth's eccentricity. a–b, MFN Mn-Al contents (this study) *versus* Earth's eccentricity and the squared wavelet coherence (WTC) (*89*) on the original timescale (*15*), using the paleomagnetic age constraints (table S3). The WTCs were conducted based on the unfiltered element data. The thin dark blue lines in all panels are the band-passed variation (80−120 kyr). See also caption in fig. S2. **c–d,** and **e–f,** results as in **a–b** but on the Mn-based and the Al-based tuning timescales, respectively. The thick black contour in the WTCs designates the 5% significance level against red noise; and the cone of influence where edge effects may distort the results are shown in a lighter shade. Arrows pointing left indicate an anti-phase relationship, and an in-phase relationship when pointing right.

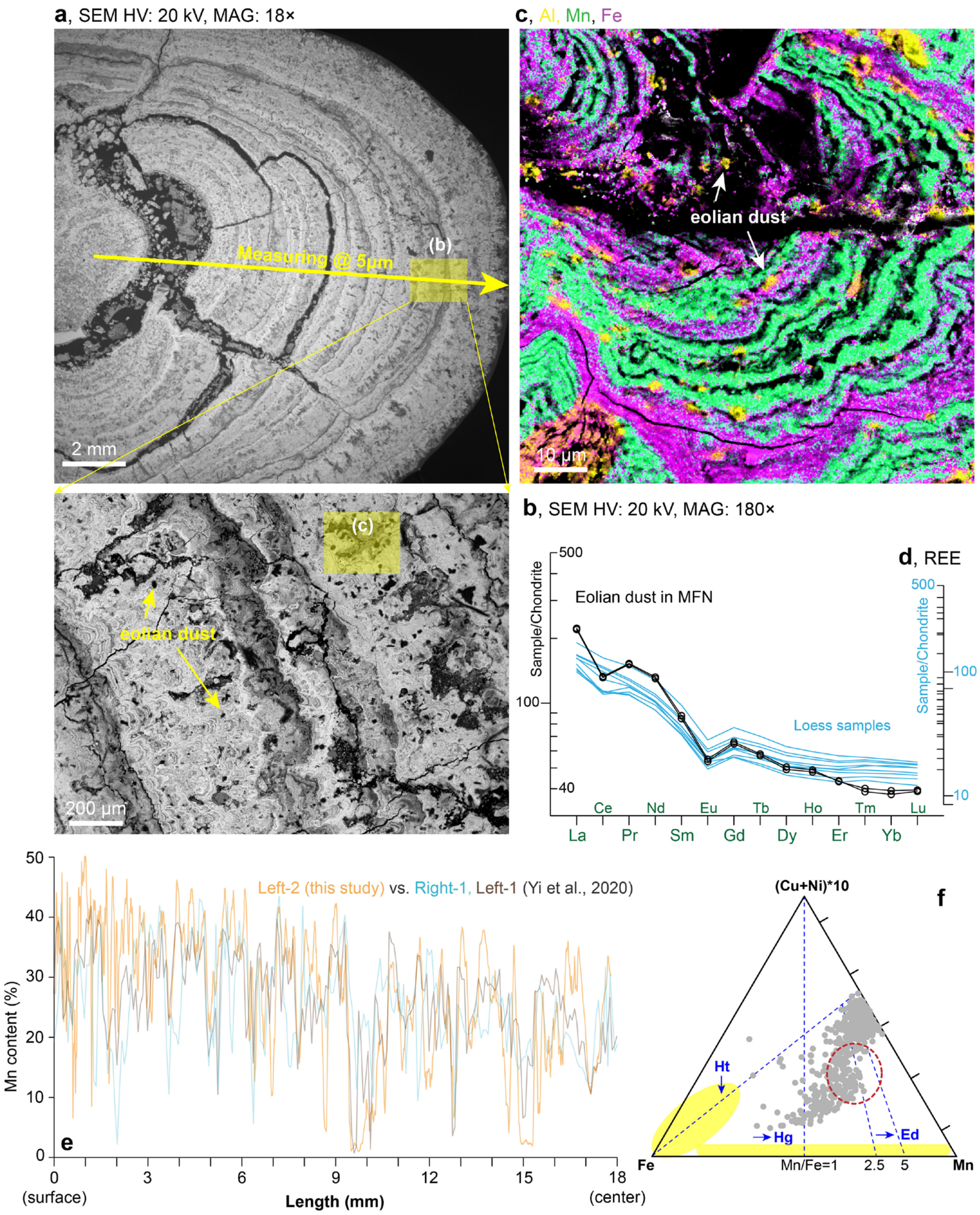

Fig. S5 SEM and element distribution of the studied MFN. a-b, SEM map of a studied section (left side), showing the measuring line in this study and eolian particles. **c**, Element distribution of Al, Mn and Fe, showing stratal configuration of Mn and Fe and random distribution of Al. **d**, Rare Earth Element (REE) data and its comparison with REE data of Chinese loess. REE data was standardized by Chondrite, which is from Taylor and McLennan (*90*); loess data are from Zhang et al. (*91*),

including the Onqin Daga, Horqin and Hunlun Buir sandy lands, Qaidam and Mu Us deserts, and Chinese loess plateau. **e**, Comparison of multiple measurements of Mn content. Right-1 and Left-1, Mn records of the right and left sides, respectively, with an 80-μm interval from Yi et al. (*15*); Left-2, Mn record measured in this study with a 5-μm interval and along the left side (shown in 80 μm spacing). All records are smoothed using a 9-point moving average. **f**, Ternary diagram of Fe, Mn, and (Cu + Ni) × 10 following (*92*). The yellow-shaded area indicates the main influence of hydrothermal process (Ht), and other areas represent the dominance of hydrogenic (Hg) and early diagenetic (Eg) processes. Dashed circle is from previously studied samples in the same area (*30*). Data in the ternary diagram are from Yi et al. (*15*).

The comparison of the left side between two measurements with different measuring intervals (left-1 and left-2) illustrates a good consistency between sides, demonstrating the robustness of chemical scans. After removing some small offsets and calculating the 9-point moving average for both sides, a good consistency is observed ($r = 0.61$, $p < 0.01$), underlining the symmetric growth of the studied MFN (*15*). Given the demonstrated growth symmetry, and that no abyssal storms, hyperpycnal flows, or sediment slumping was identified from the studied area (*31, 93*), as well as the similar pattern of palaeomagnetic scans from the left and right sides and demagnetization behaviors of thin slices (*15*), it is confirmed that the nodule did not roll over during its growth.

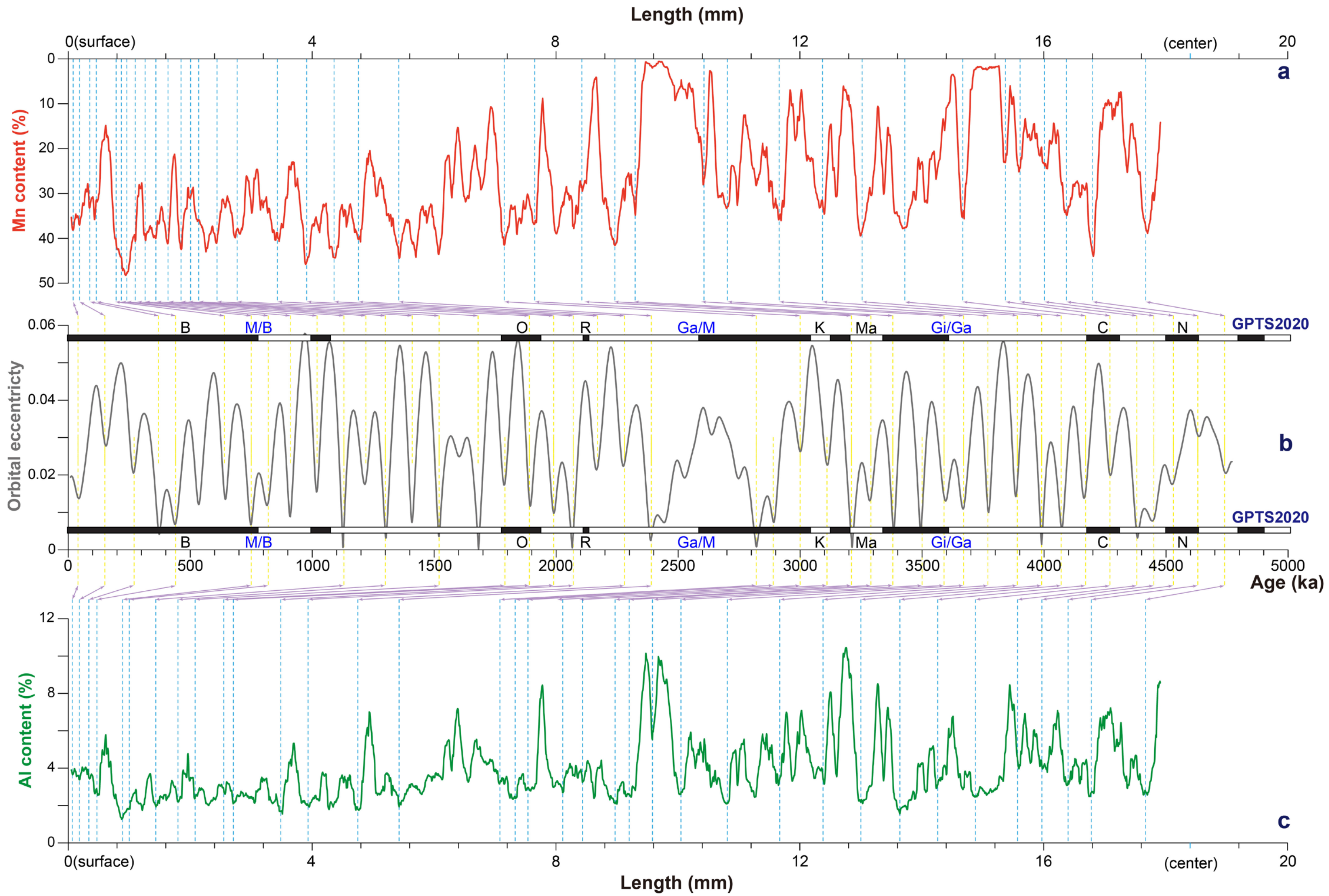

Length (mm)
0(surface)
4
8
12
16
(center)
20
Mn content (%)
a
Orbital eccentricty
b
GPTS2020
B
M/B
O
R
Ga/M
K
Ma
Gi/Ga
C
N
Age (ka)
Al content (%)
c

Fig. S6 Details of eccentricity tuning of MFN element variations. a & c, MFN Mn-Al contents (this study) *versus* Earth's eccentricity (**b**). Dash lines between records indicate the tuning points (see data in table S4). GPTS2020, Geomagnetic Polarity Time Scale (*84*). B, Brunhes; O, Olduvai; R, Réunion; K, Keana; Ma, Mammoth; C, Cochiti; N, Nunivak; M/B, Matuyama-Brunhes boundary; Ga/M, Gauss-Matuyama boundary; Gi/Ga, Gilbert-Gauss boundary.

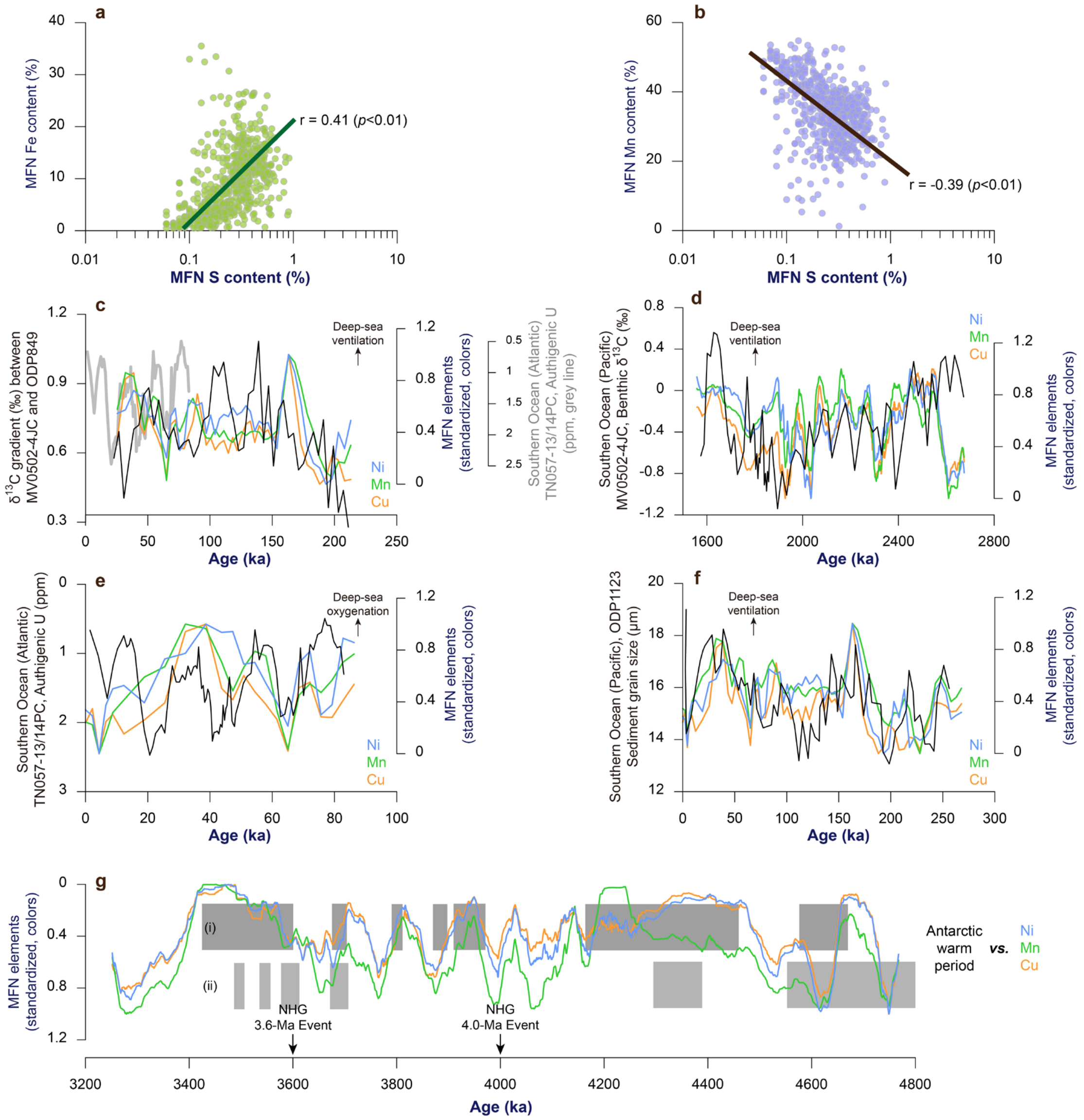

Fig. S7 Comparison of element abundance changes (Ni, Mn and Cu) of the studied MFN with various bottom water proxies. a–b, Cross plots between MFN S content and Fe and Mn contents, respectively. The S, Fe, and Mn data are from Yi et al. (*15*). The association between MFN S, Fe, and Mn demonstrates that increases in Fe content and decreases in Mn content indicate lower-oxygen conditions. **c–d,** The benthic foraminiferal $\delta^{13}C$ record of core MV0502-4JC from the Southern Ocean (Pacific), and its gradient (i.e., the difference) to the one of ODP Site 849 (Eastern Pacific), respectively, indicating deep-sea ventilation and LCDW/AABW intensity

(*34*). **e,** The authigenic uranium record of core TN057-13/14PC from the Southern Ocean (Atlantic), indicating deep-sea oxidation (*37*). **f,** The sediment grain-size record of ODP Site 1123 site, indicating deep-sea ventilation (*36*). Arrows in **c–f** show direction of enhancement of various processes. **g,** Warm periods (grey bars) around Antarctica, identified from (i) Pliocene high-productivity intervals at IODP Site U1361 (*41*), and (ii) Diatom and silicoflagellate assemblages from the Kerguelen Plateau (*82*) and Prydz Bay (*83*). Two NHG events occurred at 3.6 Ma and 4.0 Ma are identified from both marine and terrestrial records (*3, 55*). The Ni, Mn, and Cu data in **c–g** are newly obtained in this study and shown on our Mn-based timescale (see fig. S3 and table S3 for details of the timescales). The sites mentioned can be found in Fig. 1. Correlation coefficients are in table S1.

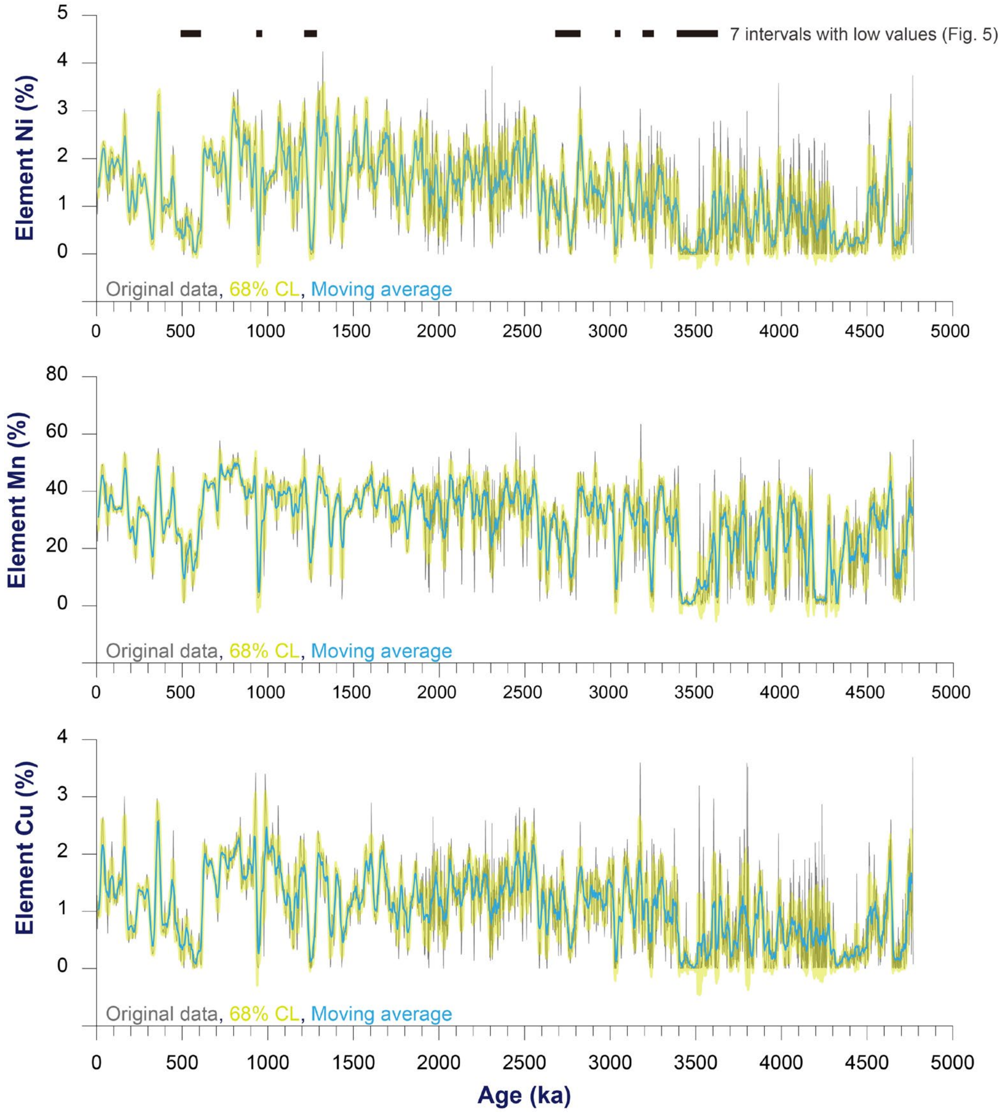

Fig. S8 Changes in MFN oxygen-sensitive metals (Ni, Mn and Cu). CL, confidence level; CL and moving average were based on 21-point (kyr) calculation. Note that CLs are based on the standard deviation of 21-point moving.

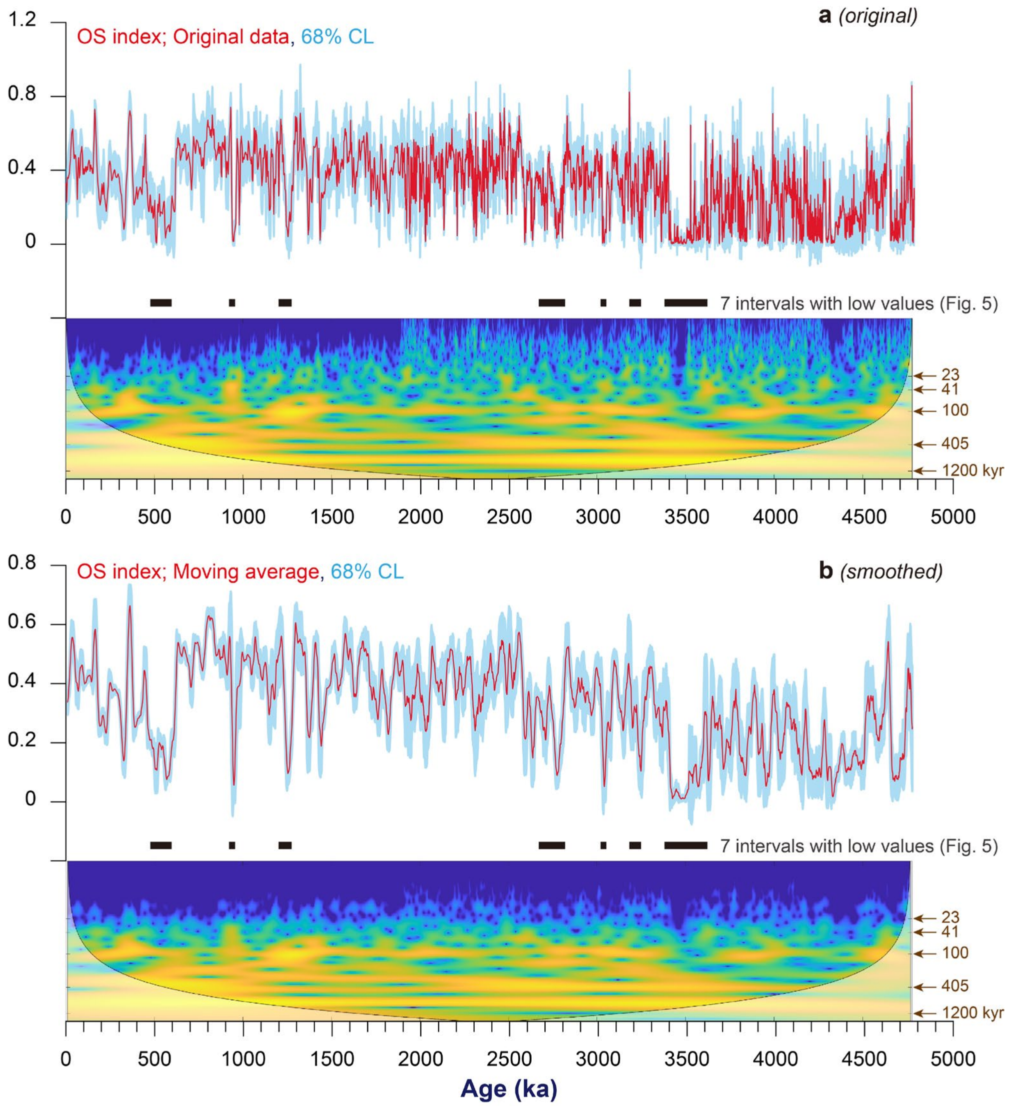

Fig. S9 The oxygen index (OS index) determined in this study from elements Ni, Mn, and Cu, with evolutionary spectra. **a**, original values; **b**, 21-point moving average. CL, confidence level; CL and moving average were based on 21-point (kyr) calculation. Note that the CL in the upper panel was from the standard deviation of three standardized elements (Ni, Mn and Cu), and CL in the lower panel is based on the standard deviation of 21-point moving.

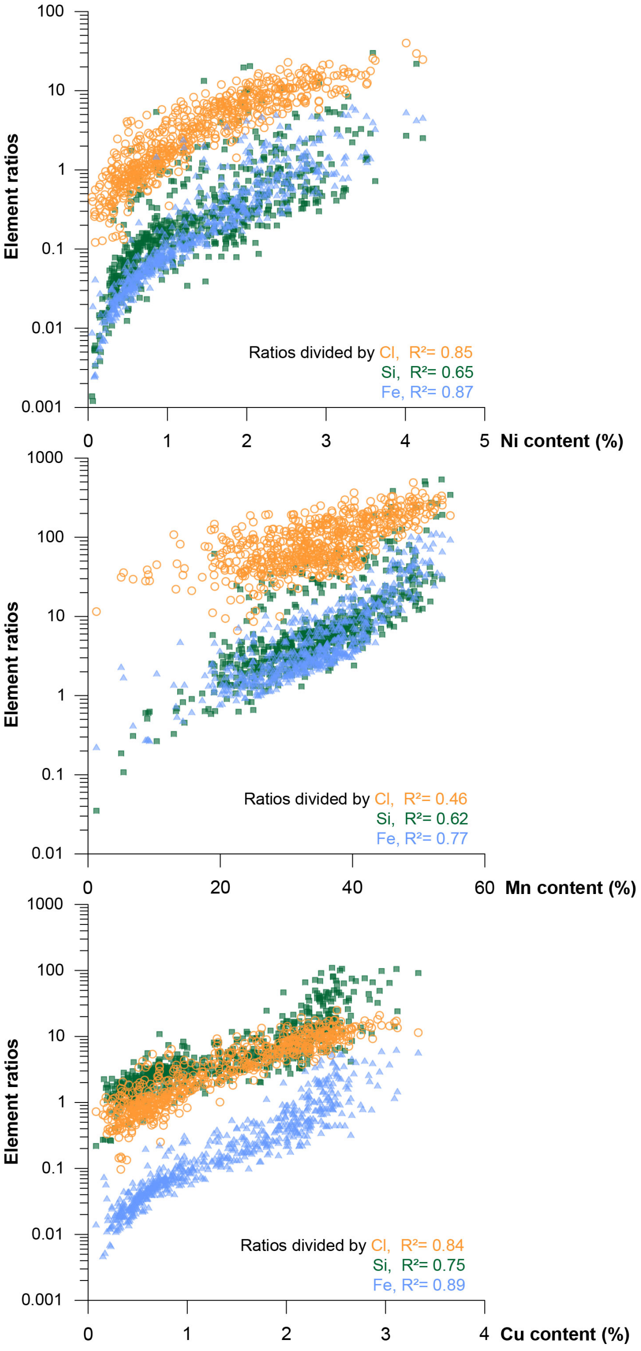

Fig. S10 Comparison between MFN metals (Ni, Mn and Cu) and their ratios. Element ratios are calculated by dividing Cl, Si, and Fe, as used in previous studies (*94-97*). All the scatter plots show a high similarity between ratios, indicating that using ratios to calculate MFN OS index has no evident difference to using the original records.

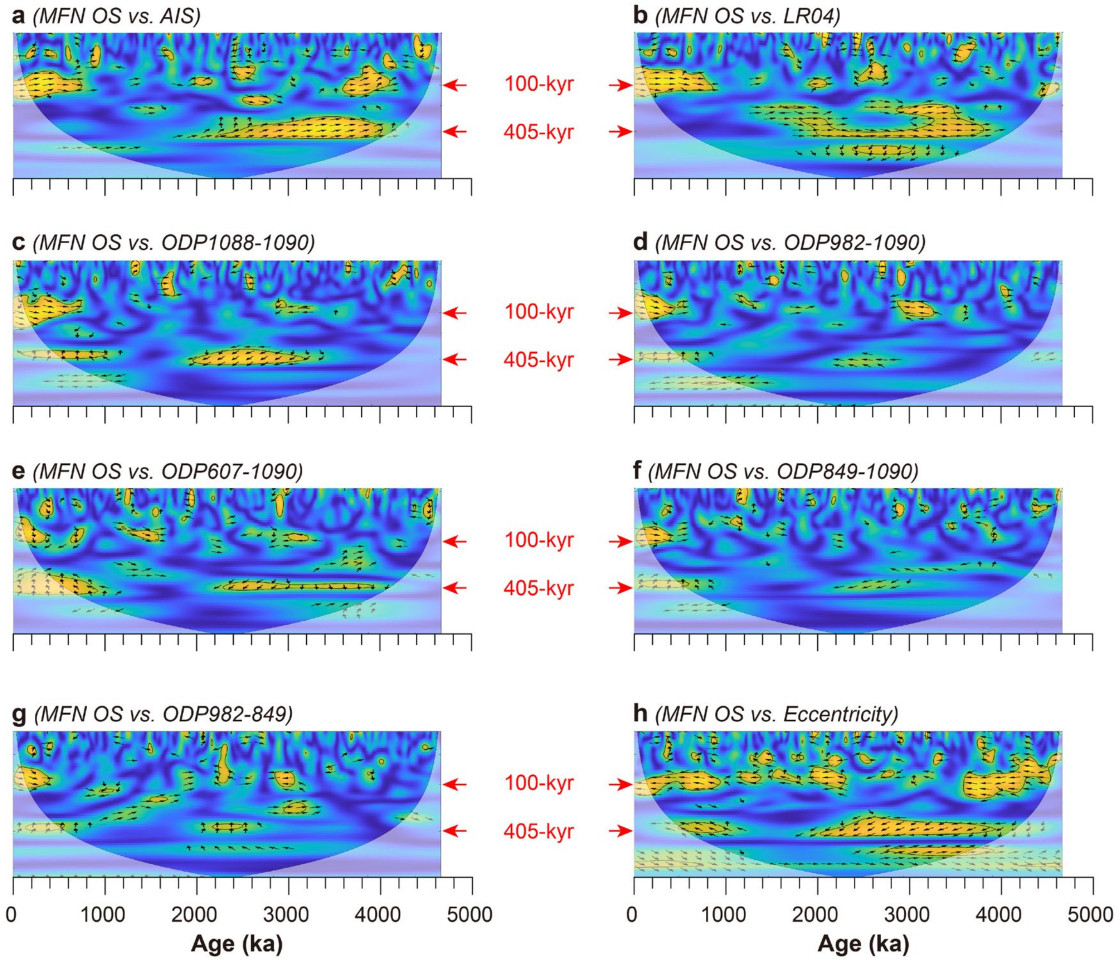

Fig. S11 Results of the squared wavelet coherence (WTC) between various proxies. The proxies are the same as **Fig. 2**. The thick black contour in the WTCs designates the 5% significance level against red noise; and the cone of influence where edge effects may distort the results is shown in a lighter shade. Arrows pointing left indicate an anti-phase relationship between eccentricity and element changes, and an in-phase relationship pointing right.

Table S1. Pearson correlation coefficients [a)] between MFN OS index and various proxies [b)] using different timescales [c)]

Timescale	Element	TN057-13/14PC (0-80 ka)	ODP1123 (0-260 ka)	MV0502-4JC/ODP849 (20-210 ka)	MV0502-4JC (1.6-2.7 Ma)
Original	Ni	-0.47	0.40	0.57	0.52
	Mn	-0.76	0.61	0.53	0.42
	Cu	-0.62	0.48	0.47	0.48
Mn-based	Ni	-0.44	0.55	0.51	0.43
	Mn	-0.67	0.63	0.55	0.33
	Cu	-0.58	0.51	0.62	0.47
Al-based	Ni	-0.55	0.42	0.49	0.37
	Mn	-0.54	0.47	0.49	0.34
	Cu	-0.27	0.49	0.35	0.42

Notes:

a) All correlation coefficients are significant at $p < 0.01$ level, with a slight lag or leading phase. Before the calculation, all records were interpolated into 1-kyr interval, yielding a corresponding sample size for correlation analyses.

b) Various proxies for past climate, see fig. S7.

c) Different timescales for the studied MFN, see fig. S6 and table S3.

Table S2. Pearson correlation coefficients [a] between MFN OS index and various proxies using different timescales [b]

Proxies [c]	Original timescale	Mn-based	Al-based
AIS	0.42, [0.16, 0.66] [d]	0.53, [0.30, 0.72]	0.48, [0.24, 0.68]
LR04	0.50, [0.25, 0.69]	0.57, [0.36, 0.75]	0.52, [0.30, 0.72]
ODP1088-1090	0.32, [0.09, 0.55]	0.35, [0.08, 0.59]	0.33, [0.08, 0.59]
ODP982-1090	0.38, [0.14, 0.60]	0.45, [0.20, 0.65]	0.42, [0.18, 0.64]
ODP607-1090	0.28, [0.01, 0.52]	0.32, [0.09, 0.56]	0.29, [0.02, 0.53]
ODP849-1090	0.33, [0.08, 0.55]	0.38, [0.12, 0.60]	0.35, [0.07, 0.60]
ODP982-849	0.27, [0.02, 0.51]	0.33, [0.10, 0.55]	0.31, [0.06, 0.54]
ODP846 C37 [e]	-0.12, [-0.35, 0.11]	-0.19, [-0.45, 0.06]	-0.17, [-0.41, 0.06]
ODP849 Barite [e]	0.33, [0.16, 0.52]	0.30, [0.11, 0.48]	0.30, [0.09, 0.49]
Eccentricity	0.07 ($p > 0.3$), [-0.07, 0.21]	-0.44, [-0.55, -0.33]	-0.32, [-0.44, -0.21]

Notes:

a) All correlation coefficients are significant at $p < 0.01$ level, except the one between MFN OS index and eccentricity on the original timescale. Before the calculation, all records were interpolated into 10-kyr interval, yielding a sample size of 455.

b) Different timescales for the studied MFN, see fig. S6 and table S3.

c) Various proxies for past climate, see Fig. 2 and fig. S11.

d) Values in [,] represent 95% confidence interval of the correlation coefficient, which were obtained by bootstrap resampling processes setting a sample size of 100 and repeating 1000 times.

e) Marine productivity of ODP Sites 846 (*39*) and 849 (*40*) in the Eastern Pacific. The correlation coefficient between these two productivity proxy records is not significant (r = -0.05, $p > 0.3$). The relationship between deep-sea oxygenation and marine productivity is expected to be negative, because of the consumption of dissolved oxygen by organic matter. This notion is consistent with the weak relationship between the MFN OS index and the marine productivity proxy (C37) at ODP Site 846 ($p < 0.02$), but not consistent with the one between the MFN OS index and marine productivity proxy (barite content) at ODP Site 849 ($p < 0.01$). In addition, the 95% confidence interval of the weak negative correlation between the MFN and ODP Site 846 crosses the origin point (r = 0), likely implying the bootstrap resampling only significant at 1-sigma level. The substantial difference between productivity records from ODP sites 846 and 849 and their weak correlation relative to the MFN OS index may be attributed to the negligible influence of surface marine productivity on abyssal organic matter content and dissolved oxygen at these deep-sea locales in the Eastern Pacific, as shown in fig. S1a.

Table S3. Time intervals of AABW collapse using different timescales [a)]

Proxies	Original timescale	Mn-based	Al-based
Ant_c1	480–620 ka	500–590 ka	500–600 ka
Ant_c2	960 ka	940–950 ka	940–950 ka
Ant_c3	1300–1330 ka	1250–1260 ka	1270–1280 ka
Ant_c4	2680–2840 ka	2630–2780 ka	2630–2790 ka
Ant_c5	3050 ka	3030–3040 ka	3050–3060 ka
Ant_c6	3190–3220 ka	3230–3240 ka	3240 ka
Ant_c7	3390–3620 ka	3410–3630 ka	3440–3620 ka

Note: a) Different timescales for the studied MFN, see figs. S3, S4, S6, and tables S4-S5.

Table S4. Magnetic age constraints on the studied MFN used in this study

GPTS2020 [a)]	Age (Ma)	Distance from Left side (mm) [b)]	Distance from Right side (mm) [b)]
SURFACE	**0**	**0**	**0**
C1n (bottom)	0.773	0.88	1.60
C1r.1n (top)	0.990		
C1r.1n (bottom)	1.070		
C2n (top)	1.770	2.48	3.92
C2n (bottom)	1.925	2.88	4.24
C2r.1n (top)	2.116	3.68	5.20
C2r.1n (bottom)	2.140	3.84	5.52
C2An.1n (top)	2.595	6.00	7.52
C2An.1n (bottom)	3.032	7.68	
C2An.2n (top)	3.116	8.32	
C2An.2n (bottom)	3.207	8.64	
C2An.3n (top)	3.330	9.12	
C2An.3n (bottom)	3.596	10.40	10.64
C3n.1n (top)	4.187	14.88	15.52
C3n.1n (bottom)	4.300		
C3n.2n (top)	4.493		
C3n.2n (bottom)	4.631	16.72	17.04
CENTER	**4.70±0.15**	**17.92**	**18.08**

Notes:

a) The geomagnetic polarity timescale (GPTS2020) (*84*).

b) The correlation of magnetic scanning to the GPTS2020 (*15*). The average growth rates are 4.20 ± 2.21 mm/Myr for the left side and 4.94 ± 4.15 mm/Myr for the right side.

Table S5. Refined age models by tuning MFN to Earth's eccentricity

No.	Length-1 (mm) [a)]	Age-1 (Ma)	Length-2 (mm)	Age-2 (Ma)
1	0.05	40	0.05	40
2	0.21	150	0.21	150
3	0.40	370	0.32	270
4	0.46	440	0.47	440
5	0.77	640	0.89	750
6	0.87	750	1.00	820
7	0.96	820	1.42	1130
8	1.13	910	1.80	1300
9	1.26	1020	2.05	1520
10	1.45	1130	2.55	1790
11	1.65	1220	2.73	1890
12	1.85	1300	3.44	1990
13	1.97	1410	3.89	2070
14	2.14	1520	4.75	2280
15	2.44	1680	5.47	2390
16	2.77	1890	7.08	2820
17	3.43	1990	7.33	2890
18	3.91	2070	7.54	3000
19	4.36	2170	8.02	3110
20	4.76	2280	8.38	3210
21	5.42	2390	8.97	3290
22	7.15	2820	9.20	3380
23	7.65	3000	9.60	3500
24	8.47	3210	10.05	3590
25	8.95	3290	10.81	3670
26	9.28	3380	11.67	3770
27	10.38	3590	12.38	3890
28	10.81	3670	13.00	3990
29	11.66	3770	13.68	4070
30	12.37	3890	14.26	4170
31	13.02	3990	14.88	4270
32	13.72	4070	15.57	4380
33	14.67	4170	15.97	4450
34	15.37	4270	16.40	4530
35	15.61	4380	16.78	4630
36	16.01	4450	17.67	4740
37	16.37	4530	–	–
38	16.80	4630	–	–
39	17.67	4740	–	–

Note:

a) The numbers -1 and -2 represent the tuning progress based on element contents of Mn and Al, respectively.

REFERENCES AND NOTES

1. P. U. Clark, R. B. Alley, D. Pollard, Northern hemisphere ice-sheet influences on global climate change. *Science* **286**, 1104–1111 (1999).

2. J. Zachos, M. Pagani, L. Sloan, E. Thomas, K. Billups, Trends, rhythms, and aberrations in global climate 65 Ma to present. *Science* **292**, 686–693 (2001).

3. L. E. Lisiecki, M. E. Raymo, A Pliocene-Pleistocene stack of 57 globally distributed benthic $\delta^{18}O$ records. *Paleoceanography* **20**, PA1003 (2005).

4. W. F. Ruddiman, Orbital insolation, ice volume, and greenhouse gases. *Quat. Sci. Revi.* **22**, 1597–1629 (2003).

5. J. Hansen, M. Sato, G. Russell, P. Kharecha, Climate sensitivity, sea level and atmospheric carbon dioxide. *Philos. Trans. Royal Soc.* **371**, 20120294 (2013).

6. N. C. Thomas, H. J. Bradbury, D. A. Hodell, Changes in North Atlantic deep-water oxygenation across the Middle Pleistocene transition. *Science* **377**, 654–659 (2022).

7. R. McKay, T. Naish, L. Carter, C. Riesselman, R. Dunbar, C. Sjunneskog, D. Winter, F. Sangiorgi, C. Warren, M. Pagani, S. Schouten, V. Willmott, R. Levy, R. DeConto, R. D. Powell, Antarctic and Southern Ocean influences on Late Pliocene global cooling. *Proc. Natl. Acad. Sci. U.S.A.* **109**, 6423–6428 (2012).

8. S. C. Woodard, Y. Rosenthal, K. G. Miller, J. D. Wright, B. K. Chiu, K. T. Lawrence, Paleoceanography. Antarctic role in Northern Hemisphere glaciation. *Science* **346**, 847–851 (2014).

9. J. R. Toggweiler, Origin of the 100,000-year timescale in Antarctic temperatures and atmospheric CO_2. *Paleoceanography* **23**, PA2211 (2008).

10. C. Basak, H. Fröllje, F. Lamy, R. Gersonde, V. Benz, R. F. Anderson, M. Molina-Kescher, K. Pahnke, Breakup of last glacial deep stratification in the South Pacific. *Science* **359**, 900–904 (2018).

11. G. C. Johnson, Quantifying Antarctic bottom water and North Atlantic deep water volumes. *J. Geophys. Res. Oceans* **113**, C05027 (2008).

12. A. L. Gordon, *Bottom Water Formation* (Oxford, ed. 2, 2001), pp. 415–421.

13. L. Mahieu, C. Lo Monaco, N. Metzl, J. Fin, C. Mignon, Variability and stability of anthropogenic CO_2 in Antarctic bottom water observed in the Indian sector of the Southern Ocean, 1978–2018. *Ocean Sci.* **16**, 1559–1576 (2020).

14. D. A. Hodell, K. A. Venz, C. D. Charles, U. S. Ninnemann, Pleistocene vertical carbon isotope and carbonate gradients in the South Atlantic sector of the Southern Ocean. *Geochem. Geophys. Geosyst.* **4**, 1–19 (2003).

15. L. Yi, M. Medina-Elizalde, G. Kletetschka, H. Yao, Q. Simon, G. A. Paterson, D. L. Bourlès, X. Deng, J. du, H. Qin, Y. Chen, Q. Xie, J. Xiao, Y. Wang, C. Andreucci, K. Keddadouche, G. Aumaître, Y. Liu, H. Wang, Z. Shen, X. Gu, T. Smith, H. Dang, Z. Jian, T. Song, H. He, C. Deng, R. Zhu, The potential of marine ferromanganese nodules from eastern Pacific as recorders of Earth's magnetic field changes during the past 4.7 myr: A geochronological study by magnetic scanning and authigenic $^{10}Be/^{9}Be$ Dating *J. Geophys. Res.* **125**, e2019JB018639 (2020).

16. M. Uematsu, R. A. Duce, J. M. Prospero, L. Chen, J. T. Merrill, R. L. McDonald, Transport of mineral aerosol from Asia over the North Pacific Ocean. *J. Geophys. Res. Oceans* **88**, 5343–5352 (1983).

17. J. R. Hein, A. Koschinsky, T. Kuhn, Deep-ocean polymetallic nodules as a resource for critical materials. *Nat. Rev. Earth Environ.* **1**, 158–169 (2020).

18. K. M. Costa, R. F. Anderson, J. F. McManus, G. Winckler, J. L. Middleton, C. H. Langmuir, Trace element (Mn, Zn, Ni, V) and authigenic uranium (aU) geochemistry reveal sedimentary redox history on the Juan de Fuca Ridge, North Pacific Ocean. *Geochim. Cosmochim. Acta* **236**, 79–98 (2018).

19. P. E. Halbach, A. Jahn, G. Cherkashov, Marine Co-Rich Ferromanganese Crust Deposits: Description and Formation, Occurrences and Distribution, Estimated World-wide Resources, in *Deep-Sea Mining: Resource Potential, Technical and Environmental Considerations* (Springer International Publishing, 2017), pp. 65–141.

20. A. Berger, M. F. Loutre, Insolation values for the climate of the last 10 million years. *Quat. Sci. Rev.* **10**, 297–317 (1991).

21. C. Liu, J. Nie, Z. Li, Q. Qiao, J. T. Abell, F. Wang, W. Xiao, Eccentricity forcing of East Asian monsoonal systems over the past 3 million years. *Proc. Natl. Acad. Sci.* **118**, e2107055118 (2021).

22. X. Feng, F. Jiang, Z. Zhang, Z. Xiong, Y. Zhong, J. Dong, T. Chen, A. Li, X. Zou, X. Shi, Long eccentricity forcing Asian dust input into the Northwestern Pacific during the early Pleistocene. *Palaeogeogr. Palaeoclimatol. Palaeoecol.* **596**, 110963 (2022).

23. A. Koschinsky, J. R. Hein, Marine ferromanganese encrustations: Archives of changing oceans. *Elements* **13**, 177–182 (2017).

24. N. S. Skornyakova, I. O. Murdmaa, Local variations in distribution and composition of ferromanganese nodules in the Clarion-Clipperton nodule province. *Mar. Geol.* **103**, 381–405 (1992).

25. J. Dymond, M. Lyle, B. Finney, D. Z. Piper, K. Murphy, R. Conard, N. Pisias, Ferromanganese nodules from MANOP sites H, S, and R-control of mineralogical and chemical composition by multiple accretionary processes. *Geochim. Cosmochim. Acta* **48**, 931–949 (1984).

26. A. Khripounoff, J.-C. Caprais, P. Crassous, J. Etoubleau, Geochemical and biological recovery of the disturbed seafloor in polymetallic nodule fields of the Clipperton-Clarion fracture zone (CCFZ) at 5,000-m depth. *Limnol. Oceanogr.* **51**, 2033–2041 (2006).

27. G. P. Glasby, Manganese: Predominant Role of Nodules and Crusts, in *Marine Geochemistry* (Springer, 2006), pp. 371–427.

28. K. Mewes, J.M. Mogollón, A. Picard, C. Rühlemann, T. Kuhn, K. Nöthen, S. Kasten, Impact of depositional and biogeochemical processes on small scale variations in nodule abundance in the Clarion-Clipperton fracture zone. *Deep-Sea Res. I Oceanogr. Res. Pap.* **91**, 125–141 (2014).

29. C. Juan, D. Van Rooij, W. De Bruycker, An assessment of bottom current controlled sedimentation in Pacific Ocean abyssal environments. *Mar. Geol.* **403**, 20–33 (2018).

30. A. V. Wegorzewski, T. Kuhn, The influence of suboxic diagenesis on the formation of manganese nodules in the Clarion Clipperton nodule belt of the Pacific Ocean. *Mar. Geol.* **357**, 123–138 (2014).

31. K. S. Jeong, J. K. Kang, K. Y. Lee, H. S. Jung, S. B. Chi, S. J. Ahn, Formation and distribution of manganese nodule deposit in the northwestern margin of Clarion-Clipperton fracture zones, northeast equatorial Pacific. *Geo-Mar. Lett.* **16**, 123–131 (1996).

32. M. Kawabe, S. Fujio, Pacific ocean circulation based on observation. *J. Oceanogr.* **66**, 389–403 (2010).

33. J. R. Hein, K. Mizell, A. Koschinsky, T. A. Conrad, Deep-ocean mineral deposits as a source of critical metals for high- and green-technology applications: Comparison with land-based resources. *Ore Geol. Rev.* **51**, 1–14 (2013).

34. L. M. Waddell, I. L. Hendy, T. C. Moore, M. W. Lyle, Ventilation of the abyssal Southern Ocean during the late Neogene: A new perspective from the subantarctic Pacific. *Paleoceanography* **24**, PA001661 (2009).

35. A. Mackensen, G. Schmiedl, Stable carbon isotopes in paleoceanography: Atmosphere, oceans, and sediments. *Earth Sci. Rev.* **197**, 102893 (2019).

36. I. R. Hall, I. N. McCave, N. J. Shackleton, G. P. Weedon, S. E. Harris, Intensified deep Pacific inflow and ventilation in Pleistocene glacial times. *Nature* **412**, 809–812 (2001).

37. S. L. Jaccard, E. D. Galbraith, A. Martínez-García, R. F. Anderson, Covariation of deep Southern Ocean oxygenation and atmospheric CO_2 through the last ice age. *Nature* **530**, 207–210 (2016).

38. D. A. Hodell, K. A. Venz-Curtis, Late Neogene history of deepwater ventilation in the Southern Ocean. *Geochem. Geophys. Geosyst.* **7**, Q09001 (2006).

39. K. T. Lawrence, Z. Liu, T. D. Herbert, Evolution of the eastern tropical Pacific through Plio-Pleistocene glaciation. *Science* **312**, 79–83 (2006).

40. Z. Ma, A. C. Ravelo, Z. Liu, L. Zhou, A. Paytan, Export production fluctuations in the eastern equatorial Pacific during the Pliocene-Pleistocene: Reconstruction using barite accumulation rates. *Paleoceanography* **30**, 1455–1469 (2015).

41. C. P. Cook, T. van de Flierdt, T. Williams, S. R. Hemming, M. Iwai, M. Kobayashi, F. J. Jimenez-Espejo, C. Escutia, J. J. González, B.K. Khim, R. M. McKay, S. Passchier, S. M. Bohaty, C. R. Riesselman, L. Tauxe, S. Sugisaki, A. L. Galindo, M. O. Patterson, F. Sangiorgi, E. L. Pierce, H. Brinkhuis, A. Klaus, A. Fehr, J. A. P. Bendle, P. K. Bijl, S. A. Carr, R. B. Dunbar, J. A. Flores, T. G. Hayden, K. Katsuki, G. S. Kong, M. Nakai, M. P. Olney, S. F. Pekar, J. Pross, U. Röhl, T. Sakai, P. K. Shrivastava, C. E. Stickley, S. Tuo, K. Welsh, M. Yamane, Dynamic behaviour of the East Antarctic ice sheet during Pliocene warmth. *Nat. Geosci.* **6**, 765–769 (2013).

42. R. McKay, Did Antarctica initiate the ice age cycles? *Science* **346**, 812–813 (2014).

43. E. J. Brook, C. Buizert, Antarctic and global climate history viewed from ice cores. *Nature* **558**, 200–208 (2018).

44. V. Brovkin, A. Ganopolski, D. Archer, S. Rahmstorf, Lowering of glacial atmospheric CO_2 in response to changes in oceanic circulation and marine biogeochemistry. *Paleoceanography* **22**, PA4202 (2007).

45. F. Pöppelmeier, M. Gutjahr, P. Blaser, H. Schulz, F. Süfke, J. Lippold, Stable Atlantic deep water mass sourcing on glacial-interglacial timescales. *Geophys. Res. Lett.* **48**, e2021GL092722 (2021).

46. W. S. Broecker, Paleocean circulation during the Last Deglaciation: A bipolar seesaw? *Paleoceanography* **13**, 119–121 (1998).

47. C. D. Hillenbrand, G. Kuhn, T. Frederichs, Record of a Mid-Pleistocene depositional anomaly in West Antarctic continental margin sediments: An indicator for ice-sheet collapse? *Quat. Sci. Rev.* **28**, 1147–1159 (2009).

48. G. Richardson, M. R. Wadley, K. J. Heywood, D. P. Stevens, H. T. Banks, Short-term climate response to a freshwater pulse in the Southern Ocean. *Geophys. Res. Lett.* **32**, L03702 (2005).

49. C. J. Fogwill, C. S. M. Turney, N. R. Golledge, D. M. Etheridge, M. Rubino, D. P. Thornton, A. Baker, J. Woodward, K. Winter, T. D. van Ommen, A. D. Moy, M. A. J. Curran, S. M. Davies, M. E. Weber, M. I. Bird, N. C. Munksgaard, L. Menviel, C. M. Rootes, B. Ellis, H. Millman, J. Vohra, A. Rivera, A. Cooper, Antarctic ice sheet discharge driven by atmosphere-ocean feedbacks at the last glacial termination. *Sci. Rep.* **7**, 39979 (2017).

50. C. S. M. Turney, R. T. Jones, S. J. Phipps, Z. Thomas, A. Hogg, A. P. Kershaw, C. J. Fogwill, J. Palmer, C. Bronk Ramsey, F. Adolphi, R. Muscheler, K. A. Hughen, R. A. Staff, M. Grosvenor, N. R. Golledge, S. O. Rasmussen, D. K. Hutchinson, S. Haberle, A. Lorrey, G. Boswijk, A. Cooper, Rapid global ocean-atmosphere response to Southern Ocean freshening during the last glacial. *Nat. Commun.* **8**, 520 (2017).

51. M. Medina-Elizalde, D. W. Lea, M. S. Fantle, Implications of seawater Mg/Ca variability for Plio-Pleistocene tropical climate reconstruction. *Earth Planet. Sci. Lett.* **269**, 585–595 (2008).

52. Q. Hao, L. Wang, F. Oldfield, Z. Guo, Extra-long interglacial in Northern Hemisphere during MISs 15-13 arising from limited extent of Arctic ice sheets in glacial MIS 14. *Sci. Rep.* **5**, 12103 (2015).

53. J. Jouzel, V. Masson-Delmotte, O. Cattani, G. Dreyfus, S. Falourd, G. Hoffmann, B. Minster, J. Nouet, J. M. Barnola, J. Chappellaz, H. Fischer, J. C. Gallet, S. Johnsen, M. Leuenberger, L. Loulergue, D. Luethi, H. Oerter, F. Parrenin, G. Raisbeck, D. Raynaud, A. Schilt, J. Schwander, E. Selmo, R. Souchez, R. Spahni, B. Stauffer, J. P. Steffensen, B. Stenni, T. F. Stocker, J. L. Tison, M. Werner, E. W. Wolff, Orbital and millennial antarctic climate variability over the past 800,000 years. *Science* **317**, 793–796 (2007).

54. E. W. Wolff, H. Fischer, F. Fundel, U. Ruth, B. Twarloh, G. C. Littot, R. Mulvaney, R. Röthlisberger, M. de Angelis, C. F. Boutron, M. Hansson, U. Jonsell, M. A. Hutterli, F. Lambert, P. Kaufmann, B. Stauffer, T. F. Stocker, J. P. Steffensen, M. Bigler, M. L. Siggaard-Andersen, R. Udisti, S. Becagli, E. Castellano, M. Severi, D. Wagenbach, C. Barbante, P. Gabrielli, V. Gaspari, Southern Ocean sea-ice extent, productivity and iron flux over the past eight glacial cycles. *Nature* **440**, 491–496 (2006).

55. S. de Schepper, P. L. Gibbard, U. Salzmann, J. Ehlers, A global synthesis of the marine and terrestrial evidence for glaciation during the Pliocene epoch. *Earth Sci. Rev.* **135**, 83–102 (2014).

56. S. G. Purkey, W. M. Smethie Jr, G. Gebbie, A. L. Gordon, R. E. Sonnerup, M. J. Warner, J. L. Bullister, A synoptic view of the ventilation and circulation of antarctic bottom water from chlorofluorocarbons and natural tracers. *Ann. Rev. Mar. Sci.* **10**, 503–527 (2018).

57. S. S. Jacobs, Bottom water production and its links with the thermohaline circulation. *Antarct. Sci.* **16**, 427–437 (2004).

58. K. I. Ohshima, Y. Fukamachi, G. D. Williams, S. Nihashi, F. Roquet, Y. Kitade, T. Tamura, D. Hirano, L. Herraiz-Borreguero, I. Field, M. Hindell, S. Aoki, M. Wakatsuchi, Antarctic bottom water production by intense sea-ice formation in the Cape Darnley polynya. *Nat. Geosci.* **6**, 235–240 (2013).

59. S. S. Jacobs, A. F. Amos, P. M. Bruchhausen, Ross sea oceanography and Antarctic bottom water formation. *Deep-Sea Res.* **17**, 935–962 (1970).

60. M. Thoma, A. Jenkins, D. Holland, S. Jacobs, Modelling circumpolar deep water intrusions on the Amundsen Sea continental shelf, Antarctica. *Geophys. Res. Lett.* **35**, L18602 (2008).

61. M. S. Dinniman, J. M. Klinck, E. E. Hofmann, Sensitivity of circumpolar deep water transport and ice shelf basal melt along the West Antarctic Peninsula to changes in the winds. *J. Climate* **25**, 4799–4816 (2012).

62. P. C. Pardo, F. F. Pérez, A. Velo, M. Gilcoto, Water masses distribution in the Southern Ocean: Improvement of an extended OMP (eOMP) analysis. *Prog. Oceanogr.* **103**, 92–105 (2012).

63. T. Kobayashi, Rapid volume reduction in Antarctic bottom water off the Adélie/George V Land coast observed by deep floats. *Deep-Sea Res. I Oceanogr. Res. Pap.* **140**, 95–117 (2018).

64. D. Pollard, R. M. DeConto, Modelling West Antarctic ice sheet growth and collapse through the past five million years. *Nature* **458**, 329–332 (2009).

65. J. R. Toggweiler, B. Samuels, Effect of sea ice on the salinity of Antarctic bottom waters. *J. Phys. Oceanogr.* **25**, 1980–1997 (1995).

66. T. Naish, R. Powell, R. Levy, G. Wilson, R. Scherer, F. Talarico, L. Krissek, F. Niessen, M. Pompilio, T. Wilson, L. Carter, R. DeConto, P. Huybers, R. McKay, D. Pollard, J. Ross, D. Winter, P. Barrett, G. Browne, R. Cody, E. Cowan, J. Crampton, G. Dunbar, N. Dunbar, F. Florindo, C. Gebhardt, I. Graham, M. Hannah, D. Hansaraj, D. Harwood, D. Helling, S. Henrys, L. Hinnov, G. Kuhn, P. Kyle, A. Läufer, P. Maffioli, D. Magens, K. Mandernack, W. McIntosh, C. Millan, R. Morin, C. Ohneiser, T. Paulsen, D. Persico, I. Raine, J. Reed, C. Riesselman, L. Sagnotti, D. Schmitt, C. Sjunneskog, P. Strong, M. Taviani, S. Vogel, T. Wilch, T. Williams, Obliquity-paced Pliocene West Antarctic ice sheet oscillations. *Nature* **458**, 322–328 (2009).

67. M. E. Weber, P. U. Clark, G. Kuhn, A. Timmermann, D. Sprenk, R. Gladstone, X. Zhang, G. Lohmann, L. Menviel, M. O. Chikamoto, T. Friedrich, C. Ohlwein, Millennial-scale variability in Antarctic ice-sheet discharge during the last deglaciation. *Nature* **510**, 134–138 (2014).

68. D. J. Hill, K. P. Bolton, A. M. Haywood, Modelled ocean changes at the Plio-Pleistocene transition driven by Antarctic ice advance. *Nat. Commun.* **8**, 14376 (2017).

69. G. H. Haug, A. Ganopolski, D. M. Sigman, A. Rosell-Mele, G. E. A. Swann, R. Tiedemann, S. L. Jaccard, J. Bollmann, M. A. Maslin, M. J. Leng, G. Eglinton, North Pacific seasonality and the glaciation of North America 2.7 million years ago. *Nature* **433**, 821–825 (2005).

70. A. J. Weaver, O. A. Saenko, P. U. Clark, J. X. Mitrovica, Meltwater pulse 1A from Antarctica as a trigger of the Bølling-Allerød warm interval. *Science* **299**, 1709–1713 (2003).

71. G. Bartoli, M. Sarnthein, M. Weinelt, H. Erlenkeuser, D. Garbe-Schönberg, D.W. Lea, Final closure of Panama and the onset of Northern Hemisphere glaciation. *Earth Planet. Sci. Lett.* **237**, 33–44 (2005).

72. N. W. Driscoll, G. H. Haug, A short circuit in thermohaline circulation: A cause for Northern Hemisphere glaciation? *Science* **282**, 436–438 (1998).

73. H. Elderfield, P. Ferretti, M. Greaves, S. Crowhurst, I. N. McCave, D. Hodell, A. M. Piotrowski, Evolution of ocean temperature and ice volume through the mid-pleistocene climate transition. *Science* **337**, 704–709 (2012).

74. G. H. Haug, R. Tiedemann, Effect of the formation of the Isthmus of Panama on Atlantic Ocean thermohaline circulation. *Nature* **393**, 673–676 (1998).

75. D. Antoine, J.-M. André, A. Morel, Oceanic primary production: 2. Estimation at global scale from satellite (Coastal Zone Color Scanner) chlorophyll. *Global Biogeochem. Cycles* **10**, 57–69 (1996).

76. P. E. Halbach, G. Friedrich, U. von Stackelberg, *The Manganese Nodule Belt of the Pacific Ocean: Geological Environment, Nodule Formation, and Mining Aspects* (Ferdinand Enke Verlag, 1988).

77. J. P. Kennett, N. D. Watkins, Deep-sea erosion and manganese nodule development in the Southeast Indian ocean. *Science* **188**, 1011–1013 (1975).

78. G. Wei, Y. Liu, X. Li, M. Chen, W. Wei, High-resolution elemental records from the South China Sea and their paleoproductivity implications. *Paleoceanography* **18**, 1054 (2003).

79. R. Lumpkin, K. Speer, Global ocean meridional overturning. *J. Phys. Oceanogr.* **37**, 2550–2562 (2007).

80. H. E. Garcia, T. P. Boyer, R. A. Locarnini, Dissolved oxygen, apparent oxygen utilization, and oxygen saturation, in *World Ocean Atlas 2013 (NOAA Atlas NESDIS 75),* S. Levitus, A. Mishonov, Eds. (Silver Spring, 2013), vol. 3.

81. P. Howell, N. Pisias, J. Ballance, J. Baughman, L. Ochs, *ARAND Time-Series Analysis Software,* (Brown University, 2006).

82. S. M. Bohaty, D. M. Harwood, Southern Ocean pliocene paleotemperature variation from high-resolution silicoflagellate biostratigraphy. *Mar. Micropaleontol.* **33**, 241–272 (1998).

83. C. Escutia, M.A. Bárcena, R.G. Lucchi, O. Romero, A.M. Ballegeer, J.J. Gonzalez, D.M. Harwood, Circum-Antarctic warming events between 4 and 3.5 Ma recorded in marine sediments from the Prydz Bay (ODP Leg 188) and the Antarctic Peninsula (ODP Leg 178) margins. *Global Planet. Change* **69**, 170–184 (2009).

84. F. M. Gradstein, J. G. Ogg, M. D. Schmitz, G. M. Ogg, *Geologic Time Scale 2020* (Elsevier, 2020).

85. D. Lüthi, M. le Floch, B. Bereiter, T. Blunier, J.M. Barnola, U. Siegenthaler, D. Raynaud, J. Jouzel, H. Fischer, K. Kawamura, T. F. Stocker, High-resolution carbon dioxide concentration record 650,000–800,000 years before present. *Nature* **453**, 379–382 (2008).

86. M. C. Kennicutt, II, D. Bromwich, D. Liggett, B. Njåstad, L. Peck, S. R. Rintoul, C. Ritz, M. J. Siegert, A. Aitken, C. M. Brooks, J. Cassano, S. Chaturvedi, D. Chen, K. Dodds, N. R. Golledge, C. le Bohec, M. Leppe, A. Murray, P. C. Nath, M. N. Raphael, M. Rogan-Finnemore, D. M. Schroeder, L. Talley, T. Travouillon, D. G. Vaughan, L. Wang, A. T. Weatherwax, H. Yang, S. L. Chown, Sustained Antarctic research: A 21st century imperative. *One Earth* **1**, 95–113 (2019).

87. J. A. Carton, G. Chepurin, X. Cao, B. Giese, A simple ocean data assimilation analysis of the global upper ocean 1950–95. Part I: Methodology. *J. Phys. Oceanogr.* **30**, 294–309 (2000).

88. J. A. Carton, B. S. Giese, A reanalysis of ocean climate using simple ocean data assimilation (SODA). *Mon. Weather Rev.* **136**, 2999–3017 (2008).

89. A. Grinsted, J. C. Moore, S. Jevrejeva, Application of the cross wavelet transform and wavelet coherence to geophysical time series. *Nonlinear Processes Geophys.* **11**, 561–566 (2004).

90. S. R. Taylor, S. M. McLennan, *The Continental Crust: Its Composition and Evolution* (Blackwell Scientific Publications, 1985).

91. W. Zhang, J.-x. Zhao, J. Chen, J. Ji, L. Liu, Binary sources of Chinese loess as revealed by trace and REE element ratios. *J. Asian Earth Sci.* **166**, 80–88 (2018).

92. E. Bonatti, T. Kraemer, H. Rydell, Classification and genesis of submarine ironmanganese deposits, in *Ferromanganese Deposits on the Ocean Floor* (NSF, US, 1972), pp. 149–166.

93. H. Wang, H. Yulin, Z. Kechao, Y. Liang, D. Xiguang, L. Guanghu, R. Jiangbo, A preliminary study on magnetostratigraphy and sedimentary evolution of piston core WPC1101 from Clarion-Clipperton fracture zones, eastern Pacific. *Earth Sci. Ed.* **44**, 1892–1905 (2014).

94. L. Yi, H. Wang, X. Deng, H. Yuan, D. Xu, H. Yao, Geochronology and geochemical properties of mid-pleistocene sediments on the Caiwei Guyot in the Northwest Pacific imply a surface-to-deep linkage. *J. Mar. Sci. Eng.* **9**, 253 (2021).

95. L. Yi, D. Xu, X. Jiang, X. Ma, Q. Ge, X. Deng, H. Wang, C. Deng, Magnetostratigraphy and authigenic $^{10}Be/^{9}Be$ dating of plio-pleistocene abyssal surficial sediments on the southern slope of Mariana Trench and sedimentary processes during the Mid-Pleistocene transition. *J. Geophys. Res. Oceans* **125**, e2020JC016250 (2020).

96. G. H. Haug, K. A. Hughen, D. M. Sigman, L. C. Peterson, U. Röhl, Southward migration of the intertropical convergence zone through the holocene. *Science* **293**, 1304–1308 (2001).

97. I. W. Croudace, R. G. Rothwell, *Micro-XRF Studies of Sediment Cores: Applications of a non-destructive tool for the environmental sciences* (Springer 2015).
